# Global, regional, and national burden of multiple sclerosis from 1990 to 2019: Findings of global burden of disease study 2019

**DOI:** 10.3389/fpubh.2023.1073278

**Published:** 2023-02-17

**Authors:** Zhen Qian, Yuancun Li, Zhiqiang Guan, Pi Guo, Ke Zheng, Yali Du, Shengjie Yin, Binyao Chen, Hongxi Wang, Jiao Jiang, Kunliang Qiu, Mingzhi Zhang

**Affiliations:** ^1^Department of Ophthalmology, Joint Shantou International Eye Center of Shantou University and The Chinese University of Hong Kong, Shantou University Medical College, Shantou, China; ^2^Department of Preventive Medicine, Shantou University Medical College, Shantou, Guangdong, China

**Keywords:** multiple sclerosis, burden, global, regional, national, age-period-cohort effects

## Abstract

**Background:**

The global rising prevalence and incidence of multiple sclerosis (MS) has been reported during the past decades. However, details regarding the evolution of MS burden have not been fully studied. This study aimed to investigate the global, regional, and national burden and temporal trends in MS incidence, deaths, and disability-adjusted life years (DALYs) from 1990 to 2019 using the age-period-cohort analysis.

**Methods:**

We performed a secondary comprehensive analysis of incidence, deaths, and DALYs of MS by calculating the estimated annual percentage change from 1990 to 2019 obtained from the Global Burden of Disease (GBD) 2019 study. The independent age, period, and birth cohort effects were evaluated by an age-period-cohort model.

**Results:**

In 2019, there were 59,345 incident MS cases and 22,439 MS deaths worldwide. The global number of incidences, deaths, and DALYs of MS followed an upward trend, whereas the age-standardized rates (ASR) slightly declined from 1990 to 2019. High socio-demographic index (SDI) regions had the highest ASR of incidences, deaths, and DALYs in 2019, while the rate of deaths and DALYs in medium SDI regions are the lowest. Six regions which include high-income North America, Western Europe, Australasia, Central Europe, and Eastern Europe had higher ASR of incidences, deaths, and DALYs than other regions in 2019. The age effect showed that the relative risks (RRs) of incidence and DALYs reached the peak at ages 30–39 and 50–59, respectively. The period effect showed that the RRs of deaths and DALYs increased with the period. The cohort effect showed that the later cohort has lower RRs of deaths and DALYs than the early cohort.

**Conclusion:**

The global cases of incidence, deaths, and DALYs of MS have all increased, whereas ASR has declined, with different trends in different regions. High SDI regions such as European countries have a substantial burden of MS. There are significant age effects for incidence, deaths, and DALYs of MS globally, and period effects and cohort effects for deaths and DALYs.

## Key messages

- What is already known on this topic:

We searched PubMed for articles published until September 2022 focusing on the incidence, deaths, and overall burden of multiple sclerosis (MS), using the terms “global burden,” “incidence,” “deaths,” “disability-adjusted life years,” “epidemiology,” “multiple sclerosis,” and “age–period–cohort analysis”. A previous study reported the global burden of MS, and its time range is from 1990 to 2016. However, an assessment of the global MS disease burden, trends, and age-period-cohort effects based on the new estimates from GBD 2019 has not been done.

- What this study adds:

This study provided a comprehensive assessment of the burden of MS at the global, regional, and country-specific levels, which included incidence, deaths, and DALYs, by age, sex, and SDI from 1990 to 2019. There are significant age effects for incidence, deaths, and DALYs of MS globally, and period effects and cohort effects for deaths and DALYs.

- How this study might affect research, practice, or policy:

High SDI regions such as European countries have a substantial burden of MS, indicating that health policymakers should take appropriate measures to reduce the MS burden. The age-period-cohort analysis contributes to interpreting temporal changes in epidemiological rates and to further analyzing the risk factors of MS.

## Introduction

Multiple sclerosis (MS) is a chronic inflammatory demyelinating disease of the central nervous system that may cause neurological dysfunction in young adults (1–3). It has been reported that MS ranked the 10th leading cause of disease burden (4). Previous studies have shown that MS has a genetic susceptibility and is also associated with several environmental risk factors including Epstein-Barr virus infection, vitamin D insufficiency, smoking, and childhood obesity (5–8). However, the exact etiology of the disease is still not fully understood.

The distribution of MS varies substantially among regions. Studies have shown that Western Europe and North America have the highest prevalence, followed by Central and Eastern Europe, the Balkans, Australia, and New Zealand, while Asia, Africa, and the Middle East have the lowest prevalence (9, 10). During the past decades, previous studies have reported the increasing incidence and prevalence of MS globally (9–13). More importantly, the significantly increasing trend of MS incidence and prevalence was observed in regions considered low-prevalence areas such as India (14), Latin America (15, 16), Iran (10, 17), Japan (18), the Greater Hobart cohort of Tasmania, and Australia (19).

Over recent decades, there have been changes in the risk factors of MS contributing to the increase in MS incidence and prevalence. Previously, significant period and cohort effects have been reported for the increasing female incidence in Danish and Swiss populations (20, 21). However, evidence regarding the interactions between the effects of age, period, and cohort in MS globally is limited. Age-period-cohort (APC) analysis is an important model to investigate how and why disease trends change over time. It is a tool for interpreting temporal variables in epidemiological rates (22). The age effect represents the variations in different age groups. Period effects reflect changes over the period that affect people of all age groups simultaneously. Cohort effects are the discriminations between early and later birth cohort groups that experience the same initial exposure environment (23).

Previously, by using the 2016 GBD data, the global and regional MS burden has been reported (23). However, an assessment of the global MS disease burden, trends, and age-period-cohort effects based on the new estimates from GBD 2019 has not been done. In the current study, we aimed to demonstrate the global and regional temporal trends in MS incidence, deaths, and disability-adjusted life years from 1990 to 2019. We further investigated the effects of age, period, and cohort by using the age-period-cohort analysis.

## Method

### Study population and data resource

Annual estimates of global, regional, and national incidence, deaths, and DALYs data of MS from 1990 to 2019 were extracted from the database of the 2019 Global Burden of Diseases, Injuries, and Risk Factors (GBD) study (http://ghdx.healthdata.org/gbd-results-tool) (3). For the 2019 GBD, there are 195 countries and territories that can be categorized into five regions based on SDI quintiles from 0 (less developed) to 1 (most developed) (24): high SDI, high-middle SDI, middle SDI, low-middle SDI, and low SDI, and 21 GBD regions in terms of birth status, education, income, etc. To analyze the effect of age on incidence, death, and DALYs rate, we extracted the data of the 10 different age groups from 1 to 99 years. To compare the disease burden of MS with the other neurological disorders, we also collected data on Parkinson's disease, headache disorders, Alzheimer's disease, idiopathic epilepsy, and motor neuron disease from 2019 GBD.

### Age-standardized rates (ASR) and estimated annual percentage change (EAPC)

Age-standardized rate of incidence (ASIR), death (ASDR), and DALYs in MS were available in 2019 GBD and can be used to calculate EAPC, which can identify temporal trends of ASR relative changes of MS from 1990 to 2019 (25). We first constructed a regression linear model about ASR, i.e., *ln (ASR)* = α +β*x* +ε, where α represents intercept, β represents the slope of the fitted line, x refers to calendar year, and ε is the error term. Then we calculated EAPC as *100* × *(e*^β^ – 1) (26). ASR is considered an increasing trend if both EAPC and its lower limit of 95% confidence interval > 0; conversely, ASR is considered a decreasing trend if both EAPC and its upper limit of 95% confidence interval <0; ASR is considered to be stable along with year otherwise (27). R software (R Foundation for Statistical Computing, Vienna, Austria, version 4.1.0) was used to perform the EAPC calculation. A *p*-value of < 0.05 was considered to be statistically significant.

### Age–period–cohort model

The APC model was used to analyze the impact of three types of time-related variations—age, period, and cohort—on global incidence, death, and DALYs rate of MS. To construct the APC model, we first used the number of cases as the dependent variable and assumed that the number follows a Poisson distribution. For the incidence rate, we can construct a log-linear regression form as follows:


(1) $$\begin{array}{l} \text{Log}\left(E_{ij}\right)\;=\text{log}\left(P_{ij}\right)\;+\mu +\alpha_{i}+\beta_{j}+\gamma_{k}, \end{array}$$

where *E*_*ij*_ represents the expected incidence number in the cell (*i, j*) of MS; *P*_*ij*_ is the total exposed population for the same period; μ is the intercept or adjusted average incidence rate; α_*i*_ represents the coefficient of *i*_th_ age group; β_*j*_ represents the coefficient of the *j*_th_ period group; and γ_*k*_ represents the coefficient of the *k*_th_ cohort group (28).

Since there is multicollinearity between the age, period, and cohort, i.e., a linear correlation between any two variables, we used the intrinsic estimator (IE) method to calculate the relative coefficient. Then, the relative risks (RRs) are obtained in the exponential form of the coefficient to analyze the incidence, death, and DALYs rate of each age, period, and cohort group to the total groups (29). We used the Akaike information criterion (AIC) and the Bayesian information criterion (BIC) to evaluate the goodness of model fit, with lower AIC-value and BIC-value indicating less information loss and better goodness of fit (30, 31).

As the 95% confidence interval (CI) of parameters was unreasonably small due to the large sample size (global population) according to GBD 2019 study, to obtain a reasonable 95% confidence interval, normal distribution was used instead of Poisson distribution. The model used “rate” as the dependent variable, which was obtained by simply dividing the number of a specific group of patients by the number of exposure of the corresponding group, and presumed to follow a normal distribution with a log link function. The “apc_ie” command in STATA version 16.0 software (Stata Corp., College Station, TX, USA) was used to perform the APC analysis.

## Results

### The global incidence, deaths, and DALYs of MS and other neurological disorders

**Figure 1:**
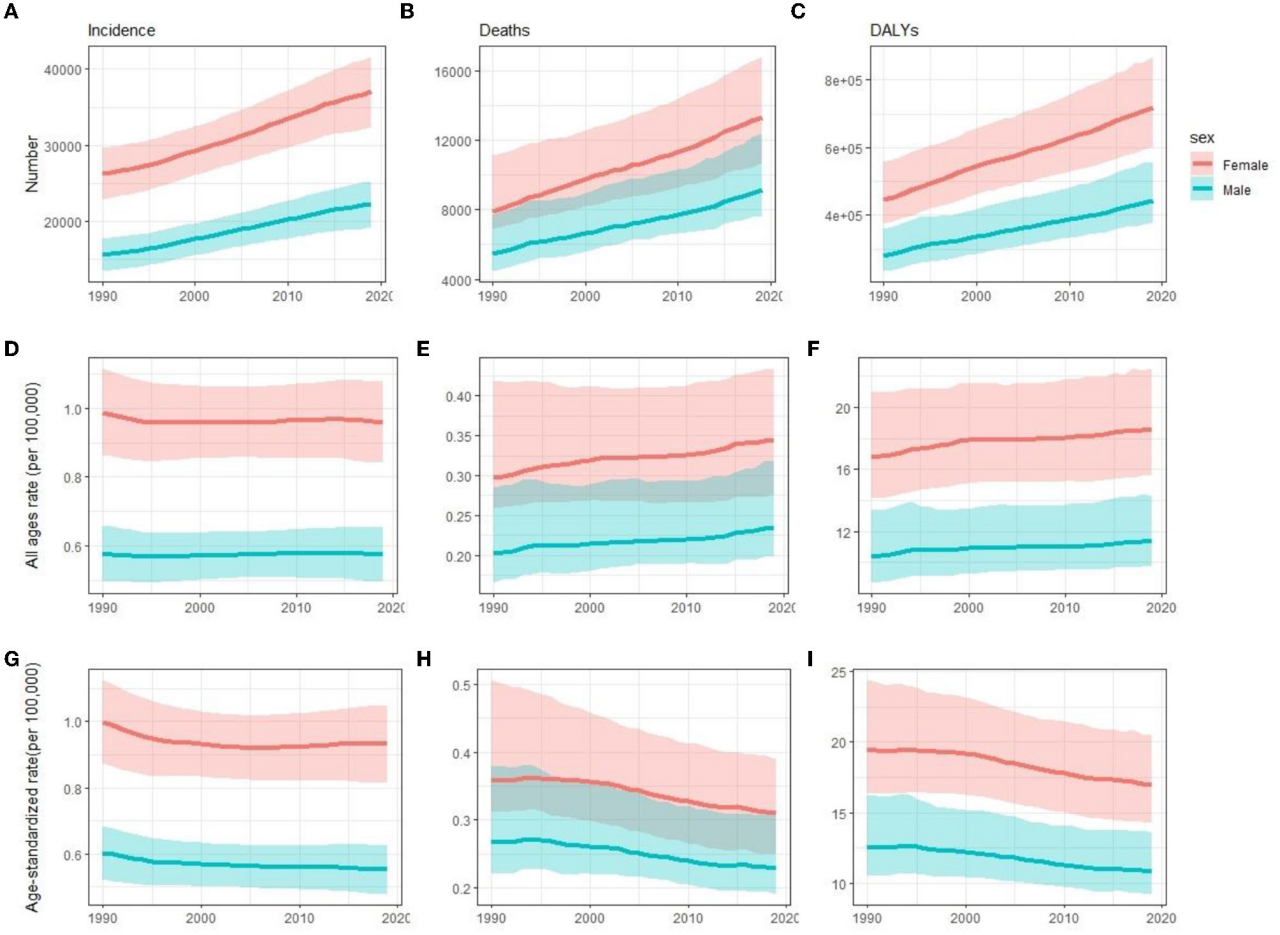
Comparison of time trends between women and men in incidence **(A, D, G)**, deaths **(B, E, H)**, and DALYs **(C, F, I)** number, all-age rate, and ASR of multiple sclerosis globally for 1990–2019, respectively. Error bars represented the 95% confidence intervals. DALYs, disability-adjusted life years; ASR, age-standardized rates.

**Figure 2:**
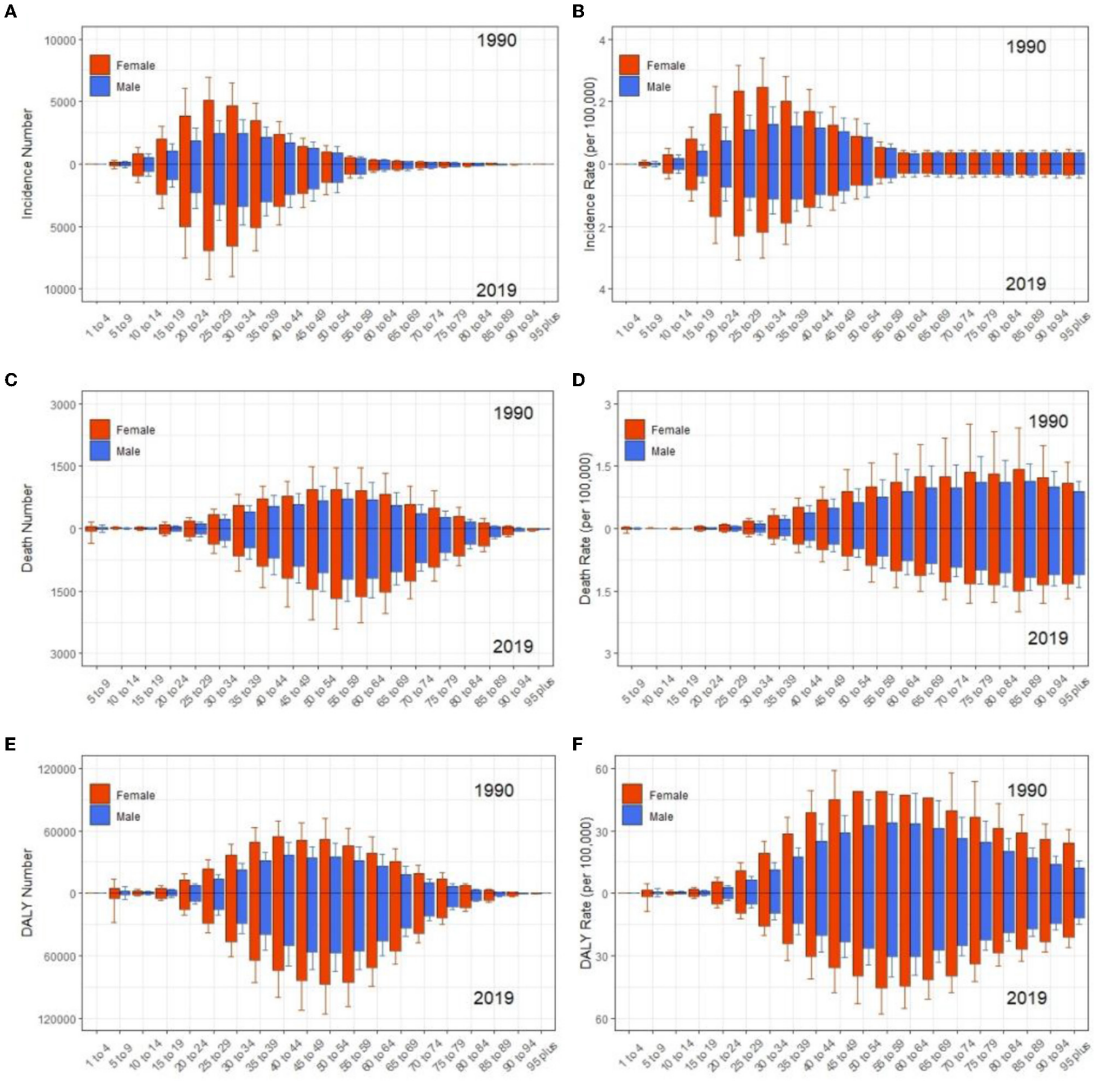
Comparison of age trends between women and men in incidence **(A, B)**, deaths **(C, D)**, and DALYs **(E, F)** number and rate of MS globally from 1990 to 2019, respectively. Error bars represented the 95% confidence intervals. DALYs, disability-adjusted life years.

At the global level, the incidence, deaths, and DALYs number increased 41.8 [from 41,854 (95% uncertainty interval [UI] 36,306.1–47,444.9) to 59,345.4 (51,817.8–66,942.6)], 68.0 [from 13,356 (11,903.5–17,571.1) to 22,439 (20,226–27,791.5)], and 59.7% [from 726,065.6 (621,892.3–867,796) to 1,159,831.8 (1,001,179.9–1,381,870.2)] from 1990 to 2019, respectively (Tables 1–3, Figures 1A–C). The incidence rate showed a relatively stable trend from 1990 to 2019, while mortality and DALYs rates presented an upward trend slightly in the same period (Figures 1D–F). However, the ASIR, ASDR, and age-standardized DALYs rates all have decreased trends in the same period, with EAPC being −0.19 (95% CI, −0.24 to −0.13), −0.62 (−0.67 to −0.56) and −0.56 (−0.6 to −0.52) (Tables 1–3, Figures 1G–I), respectively. With regard to other neurological disorders, a consistent increase both for counts and age-standardized DALYs rates was observed for Parkinson's disease [EAPC = 0.1 (95% CI, 0.04–0.16)], headache disorders [EAPC = 0.04 (0.03–0.05)], Alzheimer's disease, and other dementias [EAPC = 0.15 (0.13–0.16)] from 1990 to 2019, whereas a decrease both for counts and age-standardized DALYs rate was observed for idiopathic epilepsy [EAPC = −0.78 (−0.82 to −0.73)] and motor neuron disease [EAPC = −0.24 (−0.28 to −0.19)] (Supplementary Table 2, Supplementary Figure 1A). Overall, DALYs of MS contributed to 1.30% of all neurological disorders (Supplementary Figure 1B). Women have higher global morbidity, mortality, and, DALYs than men (Figure 2). We found that female to male ratio with MS in 2019 was greatest in the 25–29 age group and then decreased gradually for both incidence number and rate. The incidence rate peaked at the 25–29 age group in 2019 ahead of peaking at 30–34 in 1990 (Figures 2A, B).

**Table 1:** Incidence number and ASR of multiple sclerosis in 1990 and 2019, and EAPC of ASR from 1990 to 2019.

	**1990**		**2019**		**1990–2019**
	**Incidence number No. (95 UI%)**	**ASR per 100,000 No. (95 UI%)**	**Incidence number No. (95 UI%)**	**ASR per 100,000 No. (95 UI%)**	**EAPC No. × 100% (95 CI%)**
Global	41,854 (36,306.1–47,444.9)	0.8 (0.7–0.9)	59,345.4 (51,817.8–66,942.6)	0.7 (0.6–0.8)	−0.19 (−0.24 to −0.13)
**Sex**					
Male	15,614.5 (13,454.4–17,819)	0.6 (0.5–0.7)	22,329.6 (19,289.5–25,332.6)	0.6 (0.5–0.6)	−0.23 (−0.27 to −0.2)
Female	26,239.5 (22,894.8–29,645.2)	1 (0.9–1.1)	37,015.8 (32,431.3–41,581.9)	0.9 (0.8–1)	−0.16 (−0.22 to −0.09)
**SDI region**					
High SDI	20,100.6 (17,823.9–22,507.9)	2.3 (2–2.6)	24,240.3 (21,899.9–26,506.6)	2.5 (2.3–2.8)	0.4 (0.37–0.43)
High-middle SDI	10,581.3 (9,261.5–11,867.5)	0.9 (0.8–1)	12,329.8 (10,846.4–13,773.1)	0.8 (0.7–0.9)	−0.27 (−0.31 to −0.22)
Middle SDI	5,964.6 (4,919.8–7,066.4)	0.4 (0.3–0.4)	11,344 (9,469.3–13,171)	0.4 (0.4–0.5)	0.76 (0.71–0.81)
Low-middle SDI	3,647.3 (2,952.3–4,399)	0.4 (0.3–0.4)	7,448.4 (6,087.8–8,850.2)	0.4 (0.3–0.5)	0.43 (0.39–0.46)
Low SDI	1,542 (1,237.4–1,874.4)	0.4 (0.3–0.4)	3,951.4 (3,191.7–4,744.9)	0.4 (0.3–0.5)	0.32 (0.26–0.37)
**GBD region**					
Central Europe	2,246.8 (1,986.3–2,482.2)	1.8 (1.6–2)	1,840.6 (1,648.3–2,036.3)	1.7 (1.5–1.9)	−0.12 (−0.17 to −0.06)
Eastern Europe	2,920.4 (2,483.8–3,329.9)	1.3 (1.1–1.4)	2,151.7 (1,832.3–2,468.4)	1.1 (0.9–1.2)	−0.52 (−0.58 to −0.47)
Western Europe	10,253.6 (9,116–11,453.9)	2.6 (2.3–2.9)	12,497.3 (11,105.6–13,902.6)	3.2 (2.8–3.6)	0.7 (0.68–0.72)
Central Sub-Saharan Africa	93.3 (72.7–116.1)	0.2 (0.2–0.3)	243.5 (188.5–301.8)	0.2 (0.2–0.3)	0.11 (0.07–0.16)
Eastern Sub-Saharan Africa	370.3 (288.7–459.1)	0.3 (0.2–0.3)	876.2 (680.7–1,087.4)	0.3 (0.2–0.3)	0 (−0.08–0.08)
Western Sub-Saharan Africa	554.4 (445.7–668.9)	0.4 (0.3–0.4)	1,549.4 (1,264.5–1,846.1)	0.4 (0.3–0.5)	0.4 (0.39–0.42)
Southern Sub-Saharan Africa	174.2 (138.5–212.5)	0.4 (0.3–0.4)	302.2 (239.9–365.3)	0.4 (0.3–0.5)	0.08 (−0.02–0.18)
North Africa and Middle East	4,189.5 (3,598.1–4,785.4)	1.3 (1.1–1.5)	9,217.9 (7,878.6–10,525.5)	1.4 (1.2–1.6)	0.3 (0.25–0.35)
East Asia	2,100 (1,695.6–2,542.2)	0.2 (0.2–0.2)	3,119.5 (2,554.9–3,722.3)	0.2 (0.1–0.2)	−0.14 (−0.23 to −0.04)
High-income Asia Pacific	655 (528–787.4)	0.3 (0.3–0.4)	727.1 (596.5–872.5)	0.4 (0.3–0.4)	0.12 (0.09–0.16)
South Asia	3,559.5 (2,839.2–4,324)	0.4 (0.3–0.4)	7,446.5 (5,987.3–8,964.7)	0.4 (0.3–0.5)	0.33 (0.29–0.37)
Southeast Asia	724.9 (571.4–890.6)	0.2 (0.1–0.2)	1,251.5 (1,010.3–1,503.5)	0.2 (0.1–0.2)	−0.05 (−0.06 to −0.04)
Central Asia	887.5 (778.6–999.1)	1.5 (1.4–1.7)	1,351.6 (1,176.2–1,536.5)	1.4 (1.2–1.6)	−0.36 (−0.39 to −0.32)
Australasia	336.1 (300.6–373.9)	1.5 (1.4–1.7)	592.8 (519.8–664.4)	2.1 (1.8–2.3)	1.21 (0.91–1.5)
Oceania	7.5 (5.8–9.2)	0.1 (0.1–0.2)	15.8 (12.3–19.2)	0.1 (0.1–0.2)	−0.15 (−0.16 to −0.14)
Caribbean	147.7 (118.9–176.4)	0.4 (0.4–0.5)	230.3 (188.7–270.6)	0.5 (0.4–0.6)	0.26 (0.22–0.29)
Andean Latin America	85.5 (67.6–104.2)	0.3 (0.2–0.3)	206.4 (164.8–243.6)	0.3 (0.3–0.4)	0.63 (0.57–0.7)
Central Latin America	454 (361.2–548.4)	0.3 (0.3–0.4)	1,041.9 (855.9–1,222.1)	0.4 (0.3–0.5)	0.72 (0.62–0.82)
Southern Latin America	445.5 (370.4–519.2)	0.9 (0.8–1.1)	657 (550.2–760)	0.9 (0.8–1.1)	0.12 (0.12–0.13)
Tropical Latin America	1,040.6 (857.5–1,222.7)	0.7 (0.6–0.9)	1,968.4 (1,637.5–2,320.2)	0.8 (0.7–0.9)	0.25 (0.16–0.33)
High-income North America	10,607.8 (9,353.4–11,936.2)	3.5 (3.1–3.9)	12,057.8 (11,089.5–12,990.1)	3.6 (3.3–3.8)	0.24 (0.18–0.29)

ASR, age-standardized rates; EAPC, estimated annual percentage change; UI, uncertainty interval; CI, confidence interval.

**Table 2:** Deaths number and ASR of multiple sclerosis in 1990 and 2019, and EAPC of ASR from 1990 to 2019.

	**1990**		**2019**		**1990–2019**
	**Death number No. (95 UI%)**	**ASR per 100,000 No. (95 UI%)**	**Death number No. (95 UI%)**	**ASR per 100,000 No. (95 UI%)**	**EAPC No**. × **100% (95 CI%)**
Global	13,356 (11,903.5–17,571.1)	0.3 (0.3–0.4)	22,439 (20,226–27,791.5)	0.3 (0.2–0.3)	−0.62 (−0.67 to −0.56)
**Sex**					
Male	5,454.3 (4,452.4–7,679.8)	0.3 (0.2–0.4)	9,116.9 (7,670.8–12,350.5)	0.2 (0.2–0.3)	−0.66 (−0.71 to −0.61)
Female	7,901.8 (6,898–11,139.9)	0.4 (0.3–0.5)	13,322.1 (10,675–16,760.9)	0.3 (0.2–0.4)	−0.59 (−0.65 to −0.53)
**SDI region**					
High SDI	5,854.7 (5,226.9–8,068.1)	0.6 (0.5–0.8)	9,866.9 (6,992.5–11,411.2)	0.6 (0.4–0.7)	0.06 (0–0.12)
High-middle SDI	4,088.2 (3,665.6–5,644.1)	0.4 (0.3–0.5)	4,854.6 (3,918.9–7,676.4)	0.2 (0.2–0.4)	−1.72 (−1.85 to −1.59)
Middle SDI	1,791.8 (1,544.4–2,281)	0.1 (0.1–0.2)	3,839.6 (3,288.8–4,967.9)	0.1 (0.1–0.2)	−0.06 (−0.1 to −0.03)
Low-middle SDI	1,160.4 (806.4–1,666.7)	0.2 (0.1–0.2)	2,755.8 (2,319.1–3,359.2)	0.2 (0.2–0.2)	0.32 (0.27–0.38)
Low SDI	454.6 (267.8–713.3)	0.2 (0.1–0.3)	1,110.2 (829–1,461.3)	0.2 (0.1–0.2)	0.26 (0.24–0.27)
**GBD region**					
Central Europe	1,249.8 (1,052–1,548.1)	0.9 (0.7–1.1)	1,147.8 (872.5–1,844.8)	0.6 (0.5–1.1)	−1.27 (−1.35 to −1.2)
Eastern Europe	1,522.1 (1,299.8–2,260.3)	0.6 (0.5–0.9)	1,421.2 (941.8–2,847.5)	0.5 (0.3–1)	−1.31 (−1.58 to −1.04)
Western Europe	3,721.6 (3,358.6–5,327.4)	0.7 (0.6–1)	5,235.4 (3,829.1–6,538.4)	0.7 (0.5–0.9)	0 (−0.04 to 0.04)
Central Sub-Saharan Africa	30.6 (17.6–51.5)	0.1 (0.1–0.2)	78.6 (50.2–122.7)	0.1 (0.1–0.2)	0.06 (−0.02 to 0.13)
Eastern Sub-Saharan Africa	102.6 (51.3–174.8)	0.1 (0.1–0.2)	236.8 (136.1–347.9)	0.1 (0.1–0.2)	0 (−0.06 to 0.07)
Western Sub-Saharan Africa	183.6 (133–292.7)	0.2 (0.1–0.3)	559.4 (439.3–740.2)	0.2 (0.2–0.3)	1.05 (0.96–1.13)
Southern Sub-Saharan Africa	42.2 (34.9–47.8)	0.1 (0.1–0.1)	87.1 (71.4–103.7)	0.1 (0.1–0.2)	0.16 (−0.02 to 0.33)
North Africa and Middle East	580.7 (435.3–778.4)	0.3 (0.2–0.4)	1,436.3 (1,175.7–1,813.7)	0.3 (0.2–0.3)	0.16 (0.14–0.19)
East Asia	1,274.3 (923.4–1,543.1)	0.1 (0.1–0.2)	1,887.6 (1,526.7–2,506.3)	0.1 (0.1–0.1)	−1.44 (−1.59 to −1.3)
High-income Asia Pacific	237.4 (210.4–350.8)	0.1 (0.1–0.2)	321.3 (265.7–498.4)	0.1 (0.1–0.1)	−0.75 (−0.78 to −0.71)
South Asia	1,190.1 (765.7–1,753.6)	0.2 (0.1–0.3)	2,915.3 (2,386.8–3,671.7)	0.2 (0.2–0.2)	0.19 (0.11–0.26)
Southeast Asia	389.7 (324.9–536.9)	0.1 (0.1–0.2)	770.8 (592.7–1,106.5)	0.1 (0.1–0.2)	−0.36 (−0.41 to −0.3)
Central Asia	101 (81.1–121.6)	0.2 (0.2–0.2)	146.4 (117.2–222.7)	0.2 (0.2–0.3)	−0.14 (−0.29 to 0.02)
Australasia	108.7 (94.3–160)	0.5 (0.4–0.7)	214.5 (155.7–276)	0.5 (0.4–0.6)	0 (−0.08 to 0.08)
Oceania	3.7 (2.3–5.1)	0.1 (0.1–0.1)	8 (5.5–11.5)	0.1 (0.1–0.1)	−0.42 (−0.48 to −0.36)
Caribbean	59.1 (49.2–72.2)	0.2 (0.2–0.3)	122.8 (92.4–155.8)	0.2 (0.2–0.3)	0.54 (0.48–0.61)
Andean Latin America	30.6 (25.5–39.5)	0.1 (0.1–0.2)	78.7 (58.7–101)	0.1 (0.1–0.2)	0.3 (0.19–0.41)
Central Latin America	154 (132.9–240.2)	0.2 (0.1–0.2)	532.9 (414.2–682.6)	0.2 (0.2–0.3)	1.31 (1.18–1.45)
Southern Latin America	131.1 (117–185.8)	0.3 (0.3–0.4)	185 (149.9–314.7)	0.2 (0.2–0.4)	−0.82 (−0.96 to −0.69)
Tropical Latin America	158.3 (138–240.8)	0.2 (0.1–0.2)	420.6 (362.1–598.7)	0.2 (0.1–0.2)	−0.05 (−0.28 to 0.18)
High-income North America	2,084.7 (1,886.4–2,911.9)	0.6 (0.6–0.9)	4,632.5 (3,115.6–5,134.1)	0.8 (0.6–0.9)	0.69 (0.48–0.91)

ASR, age-standardized rates; EAPC, estimated annual percentage change; UI, uncertainty interval; CI, confidence interval.

**Table 3:** DALYs number and ASR of multiple sclerosis in 1990 and 2019, and EAPC of ASR from 1990 to 2019.

	**1990**		**2019**		**1990–2019**
	**DALYs number No. (95 UI%)**	**ASR per 100,000 No. (95 UI%)**	**DALYs number No. (95 UI%)**	**ASR per 100,000 No. (95 UI%)**	**EAPC No**. × **100% (95 CI%)**
Global	726,065.6 (621,892.3–867,796)	16.1 (13.8–19.3)	1,159,831.8 (1,001,179.9–1,381,870.2)	14 (12–16.6)	−0.56 (−0.6 to −0.52)
**Sex**					
Male	446,134.5 (375,656.9–557,397.5)	19.5 (16.4–24.4)	716,487.8 (603,116.4–866,743.5)	17 (14.3–20.5)	−0.54 (−0.58 to −0.5)
Female	279,931.1 (234,138.2–361,106.2)	12.6 (10.5–16.2)	443,344 (378,482.1–554,817.1)	10.8 (9.3–13.6)	−0.6 (−0.64 to −0.57)
**SDI region**					
High SDI	328,086 (277,413.6–398,197.8)	34.6 (29.2–41.9)	500,325.1 (411,436.1–581,040.5)	35.4 (29.1–41.5)	0.1 (0.05–0.16)
High-middle SDI	216,664.5 (189,121.2–269,159.3)	18.9 (16.5–23.6)	260,398.5 (207,342.4–359,882.4)	14 (11.1–19.4)	−1.33 (−1.42 to −1.23)
Middle SDI	96,587.2 (81,861.5–116,209.9)	7 (6–8.5)	199,666.1 (168,751.2–241,140.8)	7.5 (6.3–9.1)	0.21 (0.18–0.24)
Low-middle SDI	60,369.1 (44,772.5–83,402.9)	7.5 (5.6–10.2)	138,239.2 (116,847.9–167,903.7)	8.5 (7.2–10.3)	0.4 (0.35–0.44)
Low SDI	24,026.4 (16,430.2–36,047.5)	7.3 (5–10.9)	60,580.2 (47,505.3–77,711.9)	8.1 (6.4–10.2)	0.32 (0.31–0.34)
**GBD region**					
Central Europe	57,785.2 (50,040.5–67,952.8)	41.5 (35.9–48.7)	51,364 (40,324.7–75,257.2)	32.7 (25.6–48.1)	−0.95 (−1 to −0.91)
Eastern Europe	77,450.5 (65,376.4–106,470.8)	30.5 (25.9–41.6)	69,170.3 (48,216.6–125,154.3)	26.2 (18.3–47.6)	−1.14 (−1.36 to −0.91)
Western Europe	192,347 (162,402.8–237,882.1)	40.5 (34.1–49.9)	271,038.6 (222,001.5–324,345.6)	43.5 (35.8–52.7)	0.27 (0.26–0.29)
Central Sub-Saharan Africa	1,526.7 (1,033.3–2,369.3)	4.7 (3.1–7.2)	4,031.4 (2,877.9–5,786.6)	4.9 (3.5–7.1)	0.09 (0.02–0.16)
Eastern Sub-Saharan Africa	5,352.2 (3,377.6–8,358.8)	5 (3.1–7.7)	12,716.8 (8,611.1–17,088.6)	5.1 (3.4–6.9)	−0.01 (−0.07 to 0.06)
Western Sub-Saharan Africa	9,036.5 (6,974.5–13,288.5)	7.7 (6–11.4)	28,344 (22,716–35,169.6)	9.9 (8–12.5)	1 (0.93–1.07)
Southern Sub-Saharan Africa	2,379.7 (2,005.5–2,770.4)	6.5 (5.5–7.5)	4,686.1 (3,927.2–5,552.7)	6.6 (5.6–7.8)	0.06 (−0.02 to 0.14)
North Africa and Middle East	47,116.1 (36,127.9–60,785.3)	18.8 (14.7–23.2)	115,885.8 (93,053.4–144,757.9)	19.9 (16.1–24.7)	0.27 (0.24–0.3)
East Asia	54,947.3 (40,126.2–66,523.5)	5 (3.6–6)	75,174.9 (62,164.2–95,959.6)	3.8 (3.1–4.8)	−1.35 (−1.52 to −1.17)
High-income Asia Pacific	12,485 (10,440.7–16,555.6)	6.1 (5.1–8.1)	15,515.8 (12,323.7–20,968.3)	5.6 (4.4–7.7)	−0.39 (−0.42 to −0.35)
South Asia	61,498 (43,487.9–87,447.7)	7.8 (5.5–10.8)	144,077.3 (119,712.1–177,475.5)	8.6 (7.1–10.5)	0.29 (0.24–0.35)
Southeast Asia	18,650.9 (15,589.4–24,836.8)	5.1 (4.3–6.7)	32,509.9 (25,887.3–44,139.2)	4.5 (3.6–6.1)	−0.58 (−0.65 to −0.5)
Central Asia	7,574.3 (6,135.4–9,190.8)	14.4 (11.7–17.5)	12,221 (9,340.7–15,780.9)	13.8 (10.7–17.6)	−0.15 (−0.19 to −0.1)
Australasia	5,483.4 (4,570.8–7,033.5)	24.5 (20.4–31.4)	11,116 (9,023.6–13,401)	28.9 (23.3–35.3)	0.59 (0.4–0.78)
Oceania	167.7 (115.4–226.3)	3.8 (2.6–5.2)	363.2 (263.6–502.5)	3.5 (2.5–4.8)	−0.39 (−0.44 to −0.34)
Caribbean	2,979 (2,563.7–3,604.5)	10 (8.7–12.1)	5,729.5 (4,626.6–7,077.6)	11.3 (9.1–14)	0.45 (0.4–0.49)
Andean Latin America	1,494.2 (1,246.4–1,836.8)	5.7 (4.8–7)	3,734.9 (2,951.9–4,623.3)	6.1 (4.9–7.6)	0.33 (0.26–0.41)
Central Latin America	7,920.3 (6,629.8–11,288.2)	7 (5.8–10)	24,524 (20,014.5–29,813.9)	9.6 (7.9–11.7)	1.22 (1.1–1.35)
Southern Latin America	7,247.9 (6,031.8–9,269.3)	15.4 (12.8–19.7)	10,638.5 (8,314–15,021)	14 (11–19.8)	−0.42 (−0.5 to −0.35)
Tropical Latin America	10,529.2 (8,439.1–13,679.2)	9.1 (7.4–11.9)	25,311.8 (20,304.9–31,970.6)	10.1 (8.1–12.7)	0.29 (0.2–0.39)
High-income North America	142,094.4 (116,994.6–171,097.7)	45.3 (37.3–54.5)	241,677.9 (195,635–278,600.5)	49.3 (40.2–57.4)	0.29 (0.19–0.4)

DALYs, disability-adjusted life years; ASR, age-standardized rates; EAPC, estimated annual percentage change; UI, uncertainty interval; CI, confidence interval.

### The incidence, deaths, and DALYs in SDI regions and countries of MS

**Figure 3:**
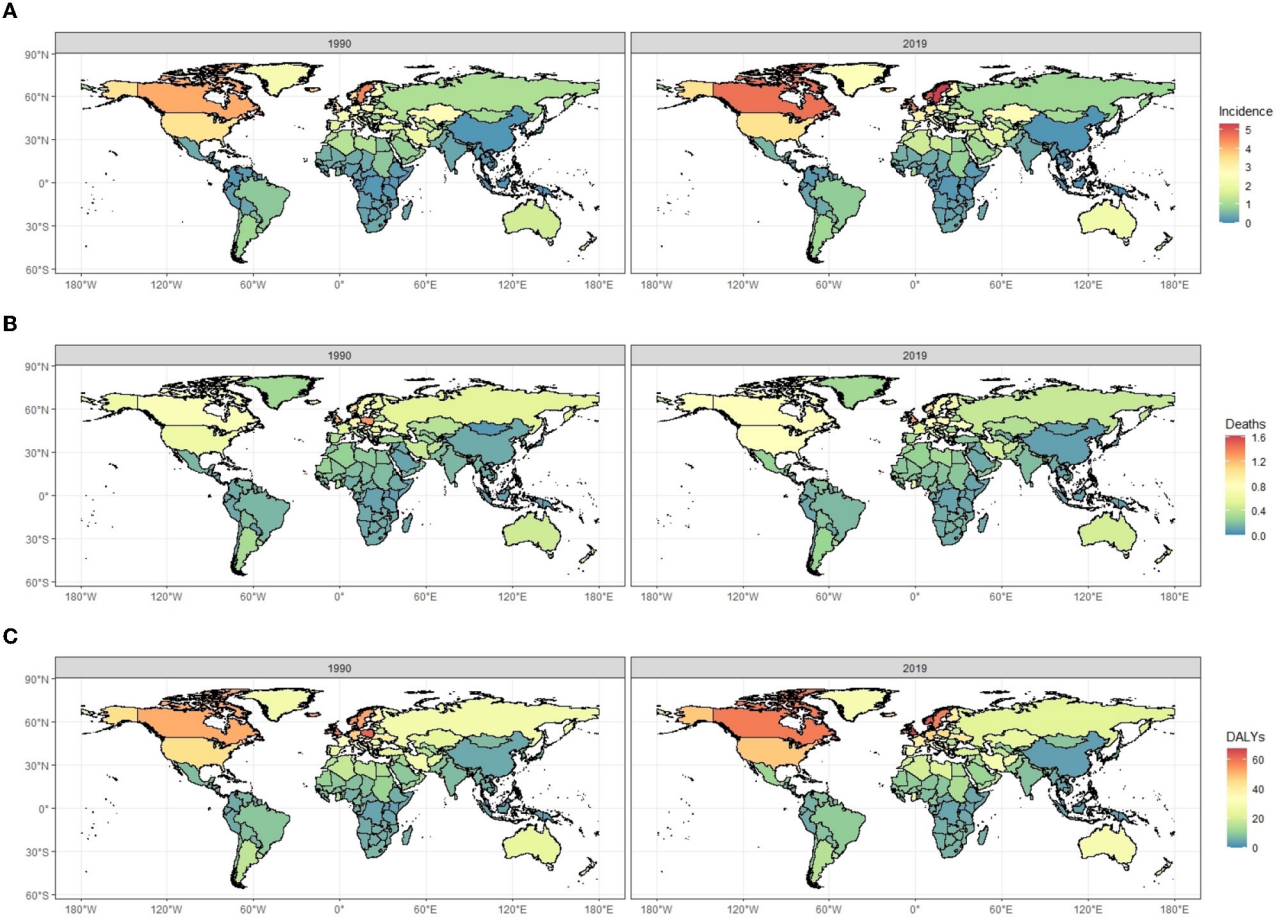
The comparisons of age-standardized incidence rates **(A)**, death rates **(B)**, and DALYs rates **(C)** of multiple sclerosis (per 100,000 population) between 1990 and 2019 for both sexes, by location. DALYs, disability-adjusted life years.

**Figure 4:**
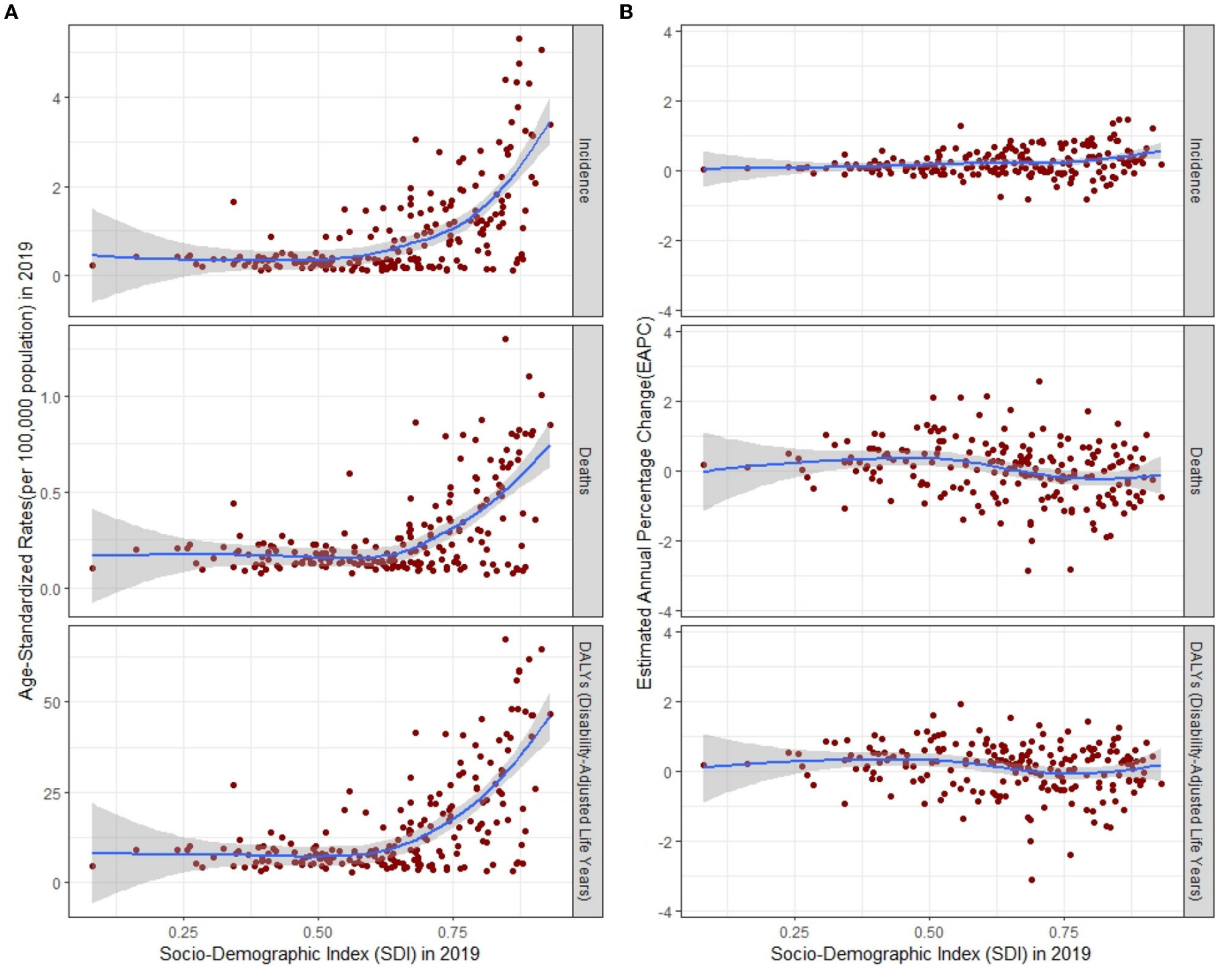
ASR **(A)** and its EAPC **(B)** of incidence, deaths, and DALYs for multiple sclerosis by SDI, 1990–2019, expected value-based SDI. The blue line represents the average expected relationship between SDI and corresponding disease burden indicator values. ASR, age-standardized rates; EAPC, estimated annual percentage change; DALYs, disability-adjusted life years; SDI, Socio-demographic Index.

Regions and countries with higher SDI had a higher number and ASR of incidence, death, and DALYs both in 1990 and 2019, while EAPC of countries from 1990 to 2019 had a low correlation with SDI (Tables 1–3, Supplementary Table 1, Figures 3, 4). ASIR increased over time in most SDI regions except the high-middle SDI region, where there was a stable decreasing trend in ASIR with EAPC is −0.27 (95% CI, −0.31 to −0.22). Among them, the ASIR of middle SDI had the highest increasing speed with EAPC being 0.76 (95% CI, 0.71–0.81) (Table 1, Figure 4). The ASDRs and ASR of DALYs of MS in medium SDI region had the lowest value in both 1990 (ASDRs = 0.1 (95% UI, 0.1–0.2)/100,000 persons, ASR of DALYs = 7 (6–8.5)/100,000 persons) and 2019 (ASDRs = 0.1 (0.1–0.2)/100,000 persons, ASR of DALYs = 7.5 (6.3–9.1)/100,000 persons). High-middle SDI had the fastest decreasing trend in ASDRs and ASR of DALYs with EAPC being −1.72 (95% CI, −1.85 to −1.59) and −1.33 (−1.42 to −1.23), respectively, while low-middle SDI had the most increasing trend with EAPC being 0.32 (0.27–0.38) and 0.4 (0.35–0.44) (Tables 2, 3, Figure 4).

### The incidence, deaths, and DALYs in GBD regions of MS

**Figure 5:**
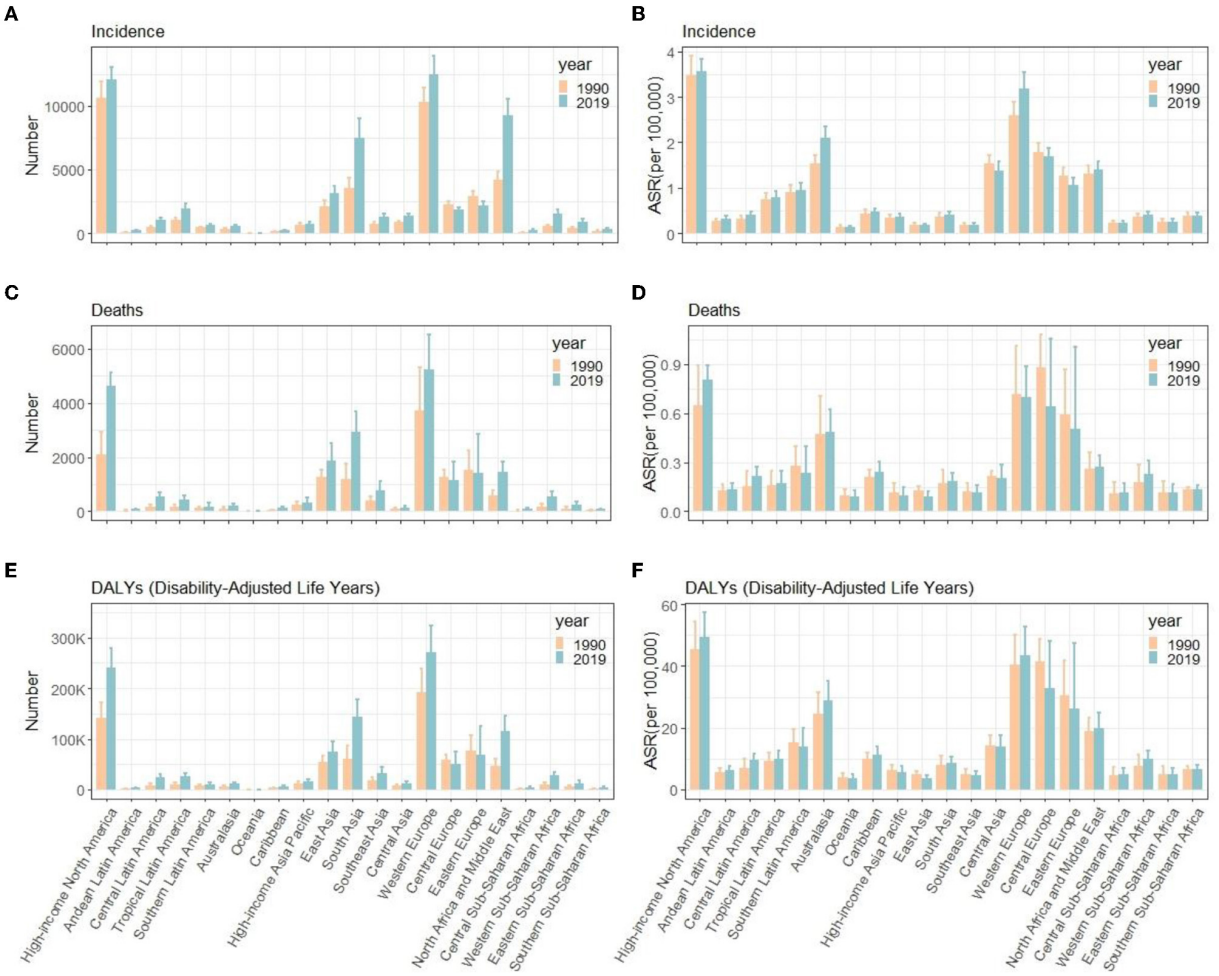
Number and ASR of incidence **(A, B)**, deaths **(C, D)**, and DALYs **(E, F)** of multiple sclerosis in different GBD countries, 1990 and 2019. ASR, age- standardized rates; DALYs, disability-adjusted life years; GBD, global burden of diseases, injuries, and risk factors study.

For the GBD region, the top two regions with the highest incidence number of MS were Western Europe [12,497.3 (95% UI, 11,105.6–13,902.6)] and high-income North America [12,057.8 (11,089.5–12,990.1)]. High-income North America [3.6 (95% UI, 3.3–3.8) per 100,000], Western Europe [0.7 (0.68–0.72) per 100,000], Australasia [1.21 (0.91–1.5) per 100,000], Central Europe [1.7 (1.5–1.9) per 100,000], and Eastern Europe [1.1 (0.9–1.2) per 100,000] had the highest ASIR of MS in 2019 than other regions. Australasia [EAPC = 1.21 (95% CI, 0.91–1.5)] and Eastern Europe [EAPC = −0.52 (−0.58 to −0.47)] had the most increase and decrease in ASIRs from 1990 to 2019, respectively (Table 1, Figures 3A, 5A, B). Regarding deaths and DALYs, absolute numbers of MS cases increased in most regions except Central Europe [1,249.8 (95% UI, 1,052–1,548.1) to 1,147.8 (872.5–1,844.8) for deaths] [57,785.2 (50,040.5–67,952.8) to 51,364 (40,324.7–75,257.2) for DALYs] and Eastern Europe [1,522.1 (1,299.8–2,260.3) to 1,421.2 (941.8–2,847.5) for deaths] [77,450.5 (65,376.4–106,470.8) to 69,170.3 (48,216.6–125,154.3) for DALYs] from 1990 to 2019. High-income North America [0.8 (95% UI, 0.6–0.9) per 100,000 persons for deaths] [49.3 (40.2–57.4) per 100,000 persons for DALYs] and Western Europe [0.7 (0.5–0.9) per 100,000 persons for deaths] [43.5 (35.8–52.7) per 100,000 persons for DALYs] were the top two regions with the highest ASDRs and ASR of DALYs in 2019. The EAPCs of ASDRs varied in different GBD regions: the most significant increasing trend was detected in Central Latin America (EAPC = 1.31, 95% CI 1.18–1.45), while East Asia (EAPC = −1.44, −1.59 to −1.3) had the most significant decrease trend. Trends in DALYs by region were broadly consistent with changes in deaths (Tables 2, 3, Figures 3B, C, 5C–F).

### Age–period–cohort analysis of MS incidence, deaths, and DALYs

**Figure 6:**
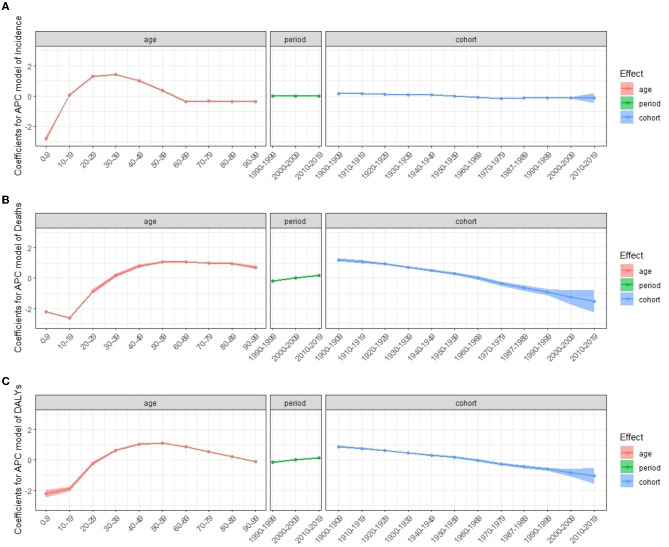
Coefficients for APC model of multiple sclerosis incidence **(A)**, deaths **(B)**, and DALYs **(C)** from 1990 to 2019. Error bars represented the 95% confidence intervals. APC, age–period–cohort; DALYs, disability-adjusted life years.

Table 4 shows the results of age–period–cohort analysis for incidence, death, and DALYs rates globally. Figure 6 shows the coefficient of MS incidence, death, and DALYs rates globally from 1990 to 2019 due to age, period, and cohort effects. After controlling for the period and cohort effects, the age effect significantly impacts the MS incidence, death, and DALYs rates. The RRs for incidence rate rise until age 30–39, then decline and plateaus after age 50–59, and the RRs for death rate and DALYs peak at 50–59 years and keep stable or decline slightly thereafter, respectively. Regarding period effect, the risk value in the death rate and DALYs had an increasing trend over time, while the incidence rate had a relatively small period effect. In terms of cohort effect, we observed decreasing trends in the risk of deaths and DALYs in later birth cohorts (Table 4, Figure 6).

**Table 4:** The age, period, and cohort effect on the global incidence, death, and DALYs rate of multiple sclerosis.

**Variable**	**Incidence**			**Deaths**			**DALYs**		
	**Coefficient (95% CI)**	**Relative risk (95% CI)**	***p*** **-value**	**Coefficient (95% CI)**	**Relative risk (95% CI)**	***p*** **-value**	**Coefficient (95% CI)**	**Relative risk (95% CI)**	***p*** **-value**
Constant	−9.36 (−9.39, −9.34)	0 (0, 0)	<0.01	−10.0 (−10.17, −10.01)	0 (0, 0)	<0.01	−6.09 (−6.14, −6.04)	0 (0, 0)	<0.01
**Age**									
0–9	−2.79 (−2.95, −2.62)	0.06 (0.05, 0.07)	<0.01	−2.23 (−2.61, −1.86)	0.11 (0.07, 0.16)	<0.01	−2.21 (−2.47, −1.96)	0.11 (0.08, 0.14)	<0.01
10–19	0.07 (0.03, 0.12)	1.08 (1.03, 1.12)	<0.01	−2.64 (−3, −2.27)	0.07 (0.05, 0.1)	<0.01	−1.9 (−2.06, −1.75)	0.15 (0.13, 0.17)	<0.01
20–29	1.32 (1.29, 1.36)	3.75 (3.62, 3.88)	<0.01	−0.86 (−1.02, −0.71)	0.42 (0.36, 0.49)	<0.01	−0.21 (−0.29, −0.12)	0.81 (0.75, 0.88)	<0.01
30–39	1.41 (1.38, 1.44)	4.09 (3.97, 4.22)	<0.01	0.16 (0.03, 0.28)	1.17 (1.03, 1.33)	0.013	0.64 (0.57, 0.7)	1.89 (1.77, 2.02)	<0.01
40–49	1.02 (1, 1.05)	2.78 (2.71, 2.85)	<0.01	0.8 (0.71, 0.9)	2.23 (2.03, 2.45)	<0.01	1.04 (0.99, 1.1)	2.84 (2.7, 2.99)	<0.01
50–59	0.36 (0.34, 0.38)	1.43 (1.4, 1.46)	<0.01	1.05 (0.98, 1.11)	2.86 (2.68, 3.05)	<0.01	1.09 (1.06, 1.13)	2.99 (2.88, 3.1)	<0.01
60–69	−0.35 (−0.37, −0.33)	0.7 (0.69, 0.72)	<0.01	1.06 (1.01, 1.1)	2.88 (2.76, 3)	<0.01	0.88 (0.86, 0.91)	2.42 (2.36, 2.48)	<0.01
70–79	−0.34 (−0.35, −0.32)	0.71 (0.7, 0.72)	<0.01	1 (0.96, 1.04)	2.72 (2.62, 2.83)	<0.01	0.56 (0.54, 0.58)	1.76 (1.72, 1.79)	<0.01
80–89	−0.34 (−0.35, −0.33)	0.71 (0.7, 0.72)	<0.01	0.95 (0.89, 1.01)	2.58 (2.43, 2.74)	<0.01	0.22 (0.19, 0.25)	1.25 (1.21, 1.29)	<0.01
**Period**									
1990–1999	0 (−0.01, 0)	1 (0.99, 1)	0.17	−0.19 (−0.22, −0.15)	0.83 (0.8, 0.86)	<0.01	−0.13 (−0.14, −0.11)	0.88 (0.87, 0.9)	<0.01
2000–2009	0 (−0.01, 0)	1 (0.99, 1)	<0.01	0 (−0.01, 0)	1 (0.99, 1)	<0.01	0 (0, 0)	1 (1, 1)	0.933
2010–2019	0.01 (0, 0.02)	1.01 (1, 1.02)	<0.01	0.19 (0.16, 0.22)	1.21 (1.17, 1.25)	<0.01	0.13 (0.11, 0.14)	1.13 (1.11, 1.15)	<0.01
**Cohort**									
1900–1909	0.19 (0.15, 0.22)	1.2 (1.17, 1.24)	<0.01	1.2 (1.09, 1.31)	3.33 (2.99, 3.71)	<0.01	0.89 (0.83, 0.95)	2.42 (2.28, 2.58)	<0.01
1910–1919	0.15 (0.13, 0.18)	1.17 (1.14, 1.2)	<0.01	1.09 (1, 1.17)	2.96 (2.73, 3.22)	<0.01	0.76 (0.71, 0.81)	2.14 (2.03, 2.24)	<0.01
1920–1929	0.13 (0.11, 0.16)	1.14 (1.11, 1.17)	<0.01	0.94 (0.87, 1)	2.56 (2.4, 2.73)	<0.01	0.63 (0.59, 0.67)	1.88 (1.8, 1.96)	<0.01
1930–1939	0.1 (0.08, 0.13)	1.11 (1.08, 1.14)	<0.01	0.7 (0.64, 0.76)	2.02 (1.91, 2.15)	<0.01	0.45 (0.41, 0.49)	1.57 (1.51, 1.64)	<0.01
1940–1949	0.07 (0.04, 0.1)	1.07 (1.04, 1.1)	<0.01	0.51 (0.43, 0.58)	1.66 (1.54, 1.78)	<0.01	0.32 (0.27, 0.37)	1.37 (1.31, 1.44)	<0.01
1950–1959	0.01 (−0.02, 0.04)	1.01 (0.98, 1.04)	0.691	0.3 (0.2, 0.39)	1.35 (1.22, 1.48)	<0.01	0.19 (0.13, 0.24)	1.2 (1.14, 1.28)	<0.01
1960–1969	−0.06 (−0.09, −0.02)	0.94 (0.91, 0.98)	<0.01	−0.01 (−0.13, 0.12)	0.99 (0.88, 1.12)	0.933	−0.02 (−0.09, 0.05)	0.98 (0.91, 1.05)	0.559
1970–1979	−0.14 (−0.17, −0.1)	0.87 (0.84, 0.9)	<0.01	−0.37 (−0.52, −0.22)	0.69 (0.59, 0.8)	<0.01	−0.28 (−0.36, −0.19)	0.76 (0.69, 0.82)	<0.01
1980–1989	−0.11 (−0.15, −0.06)	0.9 (0.86, 0.94)	<0.01	−0.64 (−0.82, −0.46)	0.53 (0.44, 0.63)	<0.01	−0.43 (−0.53, −0.33)	0.65 (0.59, 0.72)	<0.01
1990–1999	−0.12 (−0.16, −0.07)	0.89 (0.85, 0.93)	<0.01	−0.93 (−1.14, −0.72)	0.39 (0.32, 0.49)	<0.01	−0.61 (−0.73, −0.49)	0.54 (0.48, 0.61)	<0.01
2000–2009	−0.12 (−0.17, −0.06)	0.89 (0.85, 0.94)	<0.01	−1.26 (−1.73, −0.79)	0.28 (0.18, 0.45)	<0.01	−0.84 (−1.07, −0.61)	0.43 (0.34, 0.54)	<0.01
2010–2019	−0.12 (−0.43, 0.19)	0.89 (0.65, 1.21)	0.455	−1.52 (−2.27, −0.78)	0.22 (0.1, 0.46)	<0.01	−1.05 (−1.59, −0.52)	0.35 (0.2, 0.59)	<0.01
AIC	−25.31799			−26.30645			−18.13016		
BIC	−27.20958			−27.20958			−27.20958		

DALYs, disability-adjusted life years.

**Figure 7:**
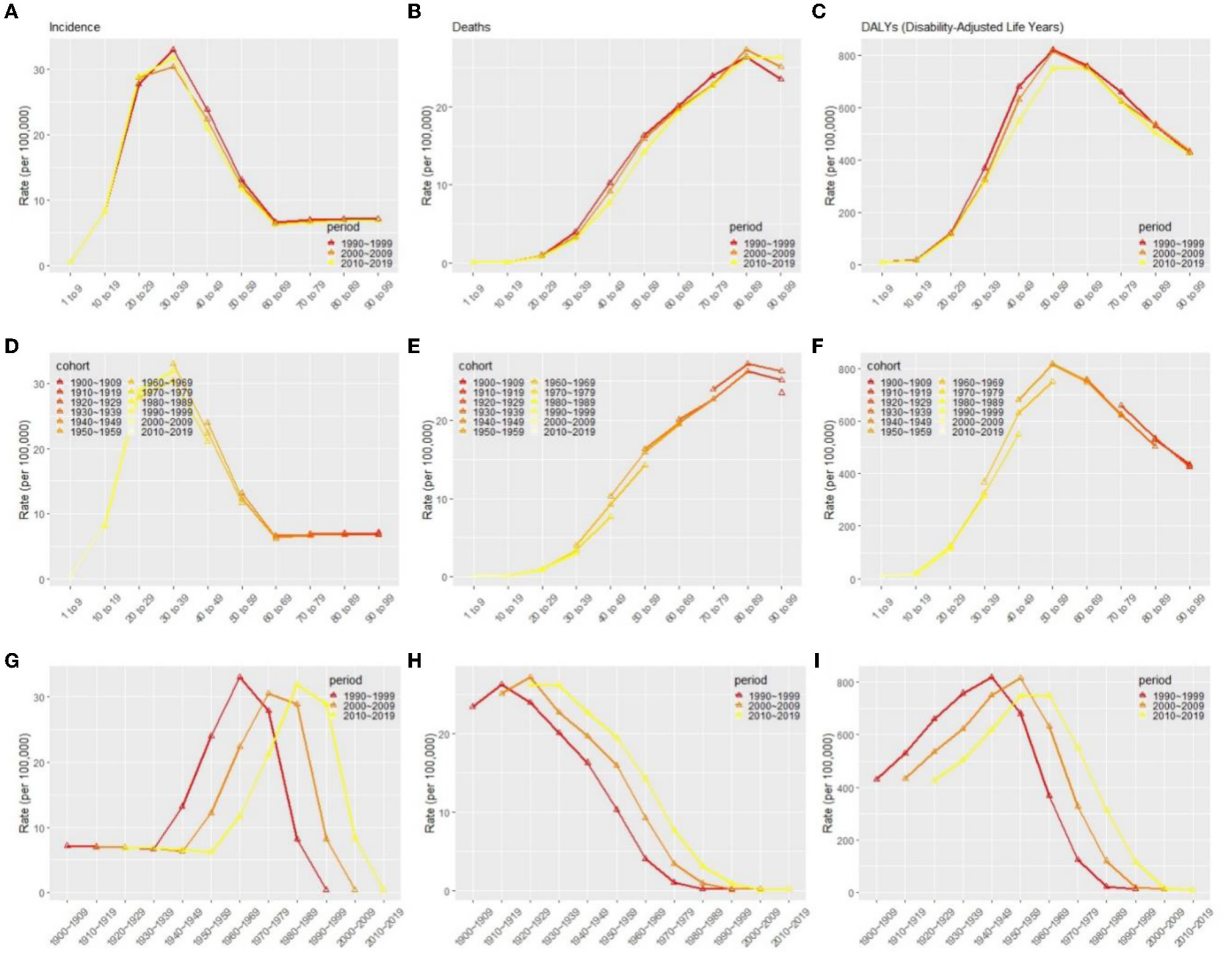
Effects of age **(A–C)**, period **(D–F)**, and cohort **(G–I)** on incidence rate, death rates, and DALYs in patients with multiple sclerosis globally, respectively. DALYs, disability-adjusted life years.

Under the influence of three temporal risk factors, the rates of incidence, death, and DALYs changed accordingly as shown in Figure 7. The incidence rate of MS in all periods increased over age and peaked at age 30–39, which then decreased and dropped to its lowest level at 60–69 (Figures 7A, D). For all periods, the death rate increased with age (Figures 7B, E), and the DALYs rate peaks at age 50–59 (Figures 7C, F). The distribution by period according to cohorts did not show significant variation (Figures 7A–C, G–I). Cohorts from 1960 to 1989 had the highest incidence rate and dropped fast afterward (Figure 7G). Death rates and DALYs rates were lower for younger generations than they were for older generations for all periods (Figures 7H, I).

## Discussion

In the current study, we evaluated the most recent examination of the global, regional, and national burden and temporal trends in MS by using the age–period–cohort analysis. From 1990 to 2019, we found that the global number of incidences, deaths, and DALYs of MS increased while the ASR decreased. The incidence rate remained relatively stable, whereas death and DALYs rates increased somewhat. Regions and countries with a higher SDI had a greater number and ASR of incidence, death, and DALYs. High-income North America and Western Europe were the top two regions with the highest ASIRs, ASDRs, and age-standardized DALYs. The age effect showed that the RRs of incidence and DALYs reached the peak at ages 30–39 and 50–59, respectively. The period effect showed that the RRs of deaths and DALYs increased with the period. The cohort effect showed that the later cohort has lower RRs of deaths and DALYs than the early cohort.

Previous studies have shown a relatively stable or slightly increasing incidence rate of MS in whites over the past four or five decades (32, 33). Our data from GBD 2019 showed that the number of incidences is increasing globally, however, the rate is flat or mildly decreasing. The combined estimate of the total MS incidence in 75 countries was 2.1 (95% CI, 2.09–2.12) per 100,000 persons/year (12), which differs from our estimate of 0.7 (95% UI, 0.6–0.8) per 100,000 persons in 2019. The lack of incidence data in some regions contributes to the discrepancy in global estimates of total incidence. We found that DALYs of MS made up 1.30% of all neurological disorders in 2019. Although the burden of MS is less than other neurological disorders such as headache disorders, Alzheimer's disease, and other dementias, the early age of MS onset and the significant impact on life quality and productivity cause a considerable non-fatal burden (11). The decreasing burden of MS, idiopathic epilepsy, and motor neuron disease observed since 1990 is partly in line with a rapidly improving quality of care, whereas an increasing burden of Parkinson's disease, Alzheimer's disease, and other dementias, which reflects increasing longevity and declining birth rates (34, 35). Several previous studies have reported a much higher crude death rate and ASDR in people with MS compared with the general population (36–38).

A positive correlation between age-standardized DALYs and SDI has been reported previously by using the GBD 2016 data (11). In agreement with the previous studies, we also detected a similar positive association between age-standardized DALY and SDI. Moreover, we found that ASIR and age-standardized DALYs in the high-medium SDI region declined significantly from 1990 to 2019 and the general improvement of clinical care and the abundance of medical resources in the regions may be the underlying causes. Our study found that ASIR from 1990 to 2019 in high SDI regions was the highest and that ASIR was moderately correlated with SDI levels, which confirmed that developed countries have a higher incidence of MS than developing countries (39). In high SDI regions, patients with MS are more likely to be treated and reported due to the availability of a robust healthcare system and adequate healthcare resources. Whereas, in low SDI regions, medical resources are so scarce that mild MS may go undetected. The significant ASIR in high SDI regions may account for its high DALYs rates. At the same time, the overall burden of MS may be much heavier than we estimated, given that data collection is limited to areas with underdeveloped health systems. The reason for the increased incidence trend of MS in most SDI regions may be that the immune system has undergone inappropriate changes over the past few decades in developed countries through the increasing use of healthy vaccinations and antibiotics, leaving people more vulnerable to autoimmune diseases (40).

Our results are consistent with some previous reports on the burden of MS in various regions (10, 11). The Atlas of MS has shown that Europe has the highest incidence, followed by the United States; South-Central Asia and Africa have the lowest incidence (12). An analysis of data from the Danish Multiple Sclerosis Registry showed that the incidence doubled in women between 1950 and 2009, while the increase in men was more modest. In contrast, the excess death rate among MS patients in Denmark has declined since 1950 (41). The incidence has been rising sharply in Iran between 1990 and 2017, possibly due to the growth of urbanization, which leads to changes in lifestyle, exposure to more weather pollutants, more stress, and consumption of fast food. DALYs are lower in the Middle East and Northern Africa, possibly due to the more benign course of MS disease (10). On the other hand, a study by Mansouri et al. indicated Latin America and the Caribbean showed increasing trends of incidence in recent years. Lack of vitamin D intake and genetic risk factors have been cited as the possible causes of this increasing trend (40).

Discrepancies in MS burden were also found across different age groups and genders. From 1990 to 2019, across all age groups, women had higher ASIR, ASDR, and ASR of DALYs due to MS. This result was consistent with those of previous studies carried out in Italy (42), Denmark (41), France (43), Australia (19, 44), and Norway (45), which similarly showed an increase in female incidence rates (46, 47). Gonadal hormones, lifestyle changes, lactation patterns, oral contraceptives, reduced physical activity, and increased stress may be the basis of this phenomenon (11, 47). Moreover, we found an earlier onset age for women in 2019 compared to 1990, indicating that more effort should be invested in women to combat MS.

There is an evident age effect on MS incidence and mortality as expected. Age effects explain the variation of indicators of interest in disease with age and reflect the nature of age changes (23, 48). The global high-risk age group for MS is 30–39 years old, which shows a similar age at MS onset patterns in published MS literature (9, 43, 49). Several countries, such as Kuwait (49), Newcastle, and Australia (44), show an age-specific bimodal distribution at MS onset during 1986–2011, indicating the existence of at least two age population segments at risk for MS. MS develops during the prime of life, and with the increasing aging of the global population (50), the burden on people living with MS will increase further, and the cost to society may soar in future. This finding broadly supports the previous study in this area that DALYs of MS globally peaked in the sixth decade (11). Higher life expectancy and early onset age have resulted in a high number of DALYs.

Although the global period effect and cohort effect of MS incidence have not been reported, the period effect and cohort effect have been evaluated in different regions previously. The period effect and cohort effect showed no significant change in the incidence of MS globally among the RRs of the different period groups and the same results showed in Lorraine, France between 1996 and 2015 (43), implying that genetic and environmental factors (5–8) that influence the disease's risk have not changed significantly or that factors that mitigate the disease's onset have emerged that have not yet been detected. On the other hand, Denmark and Kuwait show a significant period effect and cohort effect in incidence (41, 49). Moreover, a study in Norway found that the period effect on mortality was stable in men in the last three decades but increased for women (45). However, the period effect of mortality in Spain is decreasing from 1951 to 1997, probably due to an increasing life expectancy, while the risk of the birth cohort showed an increasing trend (51). A cohort effect analysis of MS-related mortality in North America and several European countries also showed a decline after the 1910–1930 generations (52). In the current study, the period effect showed that the RRs of deaths and DALYs increased with the period. The cohort effect showed that the later cohort has lower RRs of deaths and DALYs than the early cohort. Over the birth cohorts covered in our dataset, changes in lifestyle and environmental factors may have changed the risk of one cohort group over another. The late birth cohort, in comparison to the early birth cohort, received greater education, had a higher degree of awareness about health and illness prevention, and was more actively involved in treatment (22, 53). Furthermore, increasing risk factors for MS were discovered over time, raising public awareness of the disease. In APC analyses, birth cohort effects were largely unaffected by period effects due to changes in diagnostic criteria. In general, such changes are more likely too vague underlying birth cohort patterns than to emerge by chance (54).

One limitation of our current study is that the accuracy of our findings depends on the integrity and reliability of the GBD. However, insufficient diagnosis of diseases due to limited medical care in less developed regions and few national incidence and prevalence studies in high-income countries have led to the lack of partial data on the GBD, which in turn has resulted in biased models for predicting rates. In addition, the disease prediction models used in the database lack robust covariates for a more reliable risk assessment of the population (11). A second major limitation is that models are based on superimposed assumptions of age, period, and cohort effects. This not only creates identification problems but also led to a poor approximation of how social change occurs. Therefore, additional new models and methods are needed to test other theories of social change (23).

In conclusion, the cases of incidence, deaths, and DALYs of MS globally have all increased, whereas ASR has declined, with different trends in different regions. High SDI regions have a substantial burden of MS. Furthermore, we found significant age effects for incidence, deaths, and DALYs of MS globally, and period effects and cohort effects for deaths and DALYs. Health promotion, disease prevention, and rehabilitation should all receive significant attention, especially in high-risk areas.

## Data availability statement

The original contributions presented in the study are included in the article/Supplementary material, further inquiries can be directed to the corresponding authors.

## Author contributions

ZQ: study design and manuscript drafting. YL: data collection, data analysis, and drawing graphs. KZ, YD, SY, BC, HW, and JJ: data collection and data analysis. KQ, ZG, and MZ: study design, data analysis, results interpretation, and manuscript reviewing. All authors contributed to the review of manuscript and the final version of paper.
