# Supplementary material for: Global, regional, and national burden of multiple sclerosis from 1990 to 2019: Findings of global burden of disease study 2019
Source: Front Public Health. 2023 Feb 17;11:1073278. doi: 10.3389/fpubh.2023.1073278 (PMC9982151; doi:10.3389/fpubh.2023.1073278)
Supplement: Supplementary file 1 [file Data_Sheet_1.docx]

**Supplemental figure: (A)Age-standardized DALYs for neurological** **disorders from 1990 to 2019. (B) Contribution of various neurological disorders to overall neurological disorder burden in 2019.** DALYs of MS account for 1.30% of all neurological disorders. Neurological disorders contributed 3.84% (95% UI 2.21-6.16) of total DALYs globally.

Other neurological disorders include muscular dystrophy, Huntington’s disease, and other less common neurological disorders. Percentages represent proportion of DALYs. DALYs= disability-adjusted life years.

|  |  | Incidence | | Deaths | | DALYs | |  |
| --- | --- | --- | --- | --- | --- | --- | --- | --- |
|  |  | ASR per 100,000 in 2019  No. (95 UI%) | EAPC between 1990 and 2019  No. ×100% (95 CI%) | ASR per 100,000 in 2019  No. (95 UI%) | EAPC between 1990 and 2019  No. ×100% (95 CI%) | ASR per 100,000 in 2019  No. (95 UI%) | EAPC between 1990 and 2019  No. ×100% (95 CI%) |  |
|  | **High income** | | | | | | |  |
|  | High-income North America | | | | | | |  |
|  | Canada | 4.8 (4.6-4.9) | 0.31 (0.24-0.38) | 0.8 (0.5-1) | 0.05 (-0.15-0.26) | 58.8 (45.2-70.7) | 0.17 (0.08-0.26) |  |
|  | Greenland | 2.5 (2.1-3.1) | 0.3 (0.11-0.49) | 0.3 (0.2-0.4) | 0.37 (0.17-0.57) | 29.7 (22.2-38.2) | 0.31 (0.2-0.42) |  |
|  | United States of America | 3.4 (3.1-3.7) | 0.23 (0.16-0.29) | 0.8 (0.6-0.9) | 0.77 (0.55-0.99) | 48.2 (39.6-56.2) | 0.31 (0.2-0.41) |  |
|  | Australasia | | | | | | |  |
|  | Australia | 2.2 (1.9-2.5) | 1.37 (1.02-1.72) | 0.5 (0.4-0.6) | 0.13 (0.08-0.18) | 29.7 (23.9-36.4) | 0.76 (0.56-0.96) |  |
|  | New Zealand | 1.4 (1.2-1.6) | -0.17 (-0.2--0.14) | 0.5 (0.3-0.6) | -0.55 (-0.82--0.28) | 24.5 (18.8-29.1) | -0.41 (-0.58--0.24) |  |
|  | High-income Asia Pacific | | | | | | |  |
|  | Brunei | 0.2 (0.1-0.2) | 0.09 (-0.06-0.23) | 0.1 (0.1-0.2) | -0.02 (-0.17-0.12) | 5 (3.4-7.4) | -0.13 (-0.26--0.01) |  |
|  | Japan | 0.4 (0.3-0.4) | 0.27 (0.21-0.32) | 0.1 (0.1-0.2) | -0.6 (-0.63--0.56) | 5.8 (4.6-8) | -0.24 (-0.28--0.2) |  |
|  | Singapore | 0.1 (0.1-0.2) | -0.04 (-0.2-0.11) | 0.1 (0-0.1) | -1.21 (-1.25--1.16) | 3.2 (2.1-3.9) | -1.1 (-1.15--1.05) |  |
|  | Republic of Korea | 0.4 (0.3-0.5) | -0.06 (-0.1--0.02) | 0.1 (0.1-0.1) | -1.05 (-1.09--1.02) | 5.5 (4.4-6.9) | -0.63 (-0.65--0.61) |  |
|  | Western Europe | | | | | | |  |
|  | Andorra | 2.2 (1.9-2.5) | 0.44 (0.38-0.5) | 0.8 (0.4-1.3) | 0.37 (0.32-0.42) | 40.4 (25.3-57) | 0.31 (0.29-0.34) |  |
|  | Austria | 2.8 (2.5-3.2) | 0.85 (0.79-0.92) | 0.7 (0.4-0.8) | 1.36 (1.07-1.65) | 41.2 (31-49) | 0.96 (0.85-1.08) |  |
|  | Belgium | 2.7 (2.4-3.1) | 0.36 (0.34-0.38) | 0.6 (0.4-0.7) | -0.39 (-0.54--0.24) | 36.7 (29.5-44.1) | -0.1 (-0.19--0.01) |  |
|  | Cyprus | 1.5 (1.3-1.8) | 1.03 (0.94-1.12) | 0.4 (0.2-0.4) | 0.71 (0.52-0.91) | 22 (16.6-27.1) | 0.91 (0.78-1.03) |  |
|  | Denmark | 4.3 (3.9-4.8) | 0.33 (0.27-0.38) | 1.1 (0.7-1.3) | -0.87 (-0.97--0.77) | 61.9 (47.2-74.1) | -0.41 (-0.47--0.35) |  |
|  | Finland | 2.9 (2.7-3.1) | 0.36 (0.27-0.45) | 0.7 (0.4-0.8) | 0.29 (0.2-0.38) | 40.1 (30.5-47.8) | 0.24 (0.19-0.3) |  |
|  | France | 3 (2.7-3.3) | 0.94 (0.82-1.07) | 0.5 (0.3-0.6) | 0.09 (-0.12-0.29) | 39.5 (31.2-47.5) | 0.58 (0.39-0.77) |  |
|  | Germany | 3.1 (2.8-3.6) | 0.47 (0.42-0.52) | 0.8 (0.7-1.1) | -0.17 (-0.35-0.01) | 46.3 (37.8-58.8) | -0.03 (-0.1-0.05) |  |
|  | Greece | 1.3 (1.1-1.5) | 0.86 (0.8-0.92) | 0.5 (0.3-0.6) | 1.7 (1.52-1.89) | 26.2 (18-31.5) | 1.43 (1.32-1.55) |  |
|  | Iceland | 3.8 (3.3-4.4) | 0.13 (0.06-0.2) | 0.7 (0.5-1.1) | -0.93 (-1.05--0.82) | 47.9 (37.1-64.1) | -0.29 (-0.37--0.22) |  |
|  | Ireland | 4.3 (3.7-5) | 0.56 (0.44-0.67) | 0.8 (0.4-1) | -0.25 (-0.41--0.08) | 55.9 (41.6-68.6) | 0.2 (0.12-0.28) |  |
|  | Israel | 1.2 (1-1.5) | 0.39 (0.33-0.44) | 0.2 (0.1-0.3) | 0.46 (0.33-0.58) | 16.4 (11.7-20.2) | 0.42 (0.37-0.47) |  |
|  | Italy | 2.8 (2.4-3.3) | 0.94 (0.84-1.04) | 0.4 (0.3-0.6) | 0.2 (0.1-0.3) | 34.8 (27.4-43.4) | 0.62 (0.53-0.7) |  |
|  | Luxembourg | 3.2 (2.8-3.6) | 0.22 (0.17-0.28) | 0.8 (0.6-1.3) | -0.6 (-0.62--0.57) | 46.5 (36.8-62) | -0.25 (-0.27--0.23) |  |
|  | Malta | 1 (0.8-1.1) | 0.79 (0.62-0.95) | 0.3 (0.2-0.4) | -0.15 (-0.19--0.12) | 15.9 (12.8-21.2) | 0.36 (0.29-0.44) |  |
|  | Netherlands | 3.2 (2.8-3.7) | 0.27 (0.22-0.32) | 0.8 (0.6-1) | -0.71 (-0.86--0.56) | 47.3 (38.4-57) | -0.26 (-0.33--0.2) |  |
|  | Norway | 5.1 (4.3-5.8) | 1.23 (1.16-1.29) | 1 (0.5-1.2) | -0.27 (-0.47--0.07) | 64.5 (45.5-79.4) | 0.41 (0.29-0.54) |  |
|  | Portugal | 1.2 (1.1-1.3) | -0.09 (-0.1--0.08) | 0.3 (0.2-0.5) | -0.81 (-0.95--0.67) | 17.4 (13.8-24.5) | -0.54 (-0.62--0.46) |  |
|  | Spain | 2.6 (2.4-2.8) | 0.86 (0.73-0.99) | 0.3 (0.3-0.6) | -0.11 (-0.23-0) | 30.6 (24-39) | 0.44 (0.33-0.55) |  |
|  | Sweden | 5.3 (4.7-6) | 0.55 (0.51-0.59) | 0.7 (0.4-0.8) | 0.4 (0.27-0.52) | 58.5 (45.5-71.2) | 0.35 (0.3-0.4) |  |
|  | Switzerland | 3.4 (3-3.8) | 0.18 (0.15-0.21) | 0.9 (0.6-1.1) | -0.74 (-0.8--0.68) | 46.7 (37.9-58.3) | -0.38 (-0.42--0.34) |  |
|  | United Kingdom | 4.4 (3.9-4.9) | 0.85 (0.82-0.89) | 1.3 (1-1.6) | 0.45 (0.36-0.54) | 67.5 (57.2-82.3) | 0.55 (0.51-0.58) |  |
|  | Southern Latin America | | | | | | |  |
|  | Argentina | 1 (0.8-1.1) | 0.07 (0.02-0.11) | 0.3 (0.2-0.5) | -0.83 (-0.98--0.67) | 15.6 (11.8-23.3) | -0.47 (-0.57--0.37) |  |
|  | Chile | 0.8 (0.7-1) | 0.19 (0.08-0.3) | 0.1 (0.1-0.2) | -0.18 (-0.32--0.04) | 10 (7.7-13) | 0.03 (0-0.07) |  |
|  | Uruguay | 1 (0.8-1.1) | 0.12 (0.08-0.16) | 0.3 (0.2-0.4) | -0.12 (-0.35-0.11) | 17.4 (12.1-21.1) | -0.03 (-0.19-0.13) |  |
|  | **Central Europe, eastern Europe, and central Asia** | | | | | | |  |
|  | Eastern Europe | | | | | | |  |
|  | Belarus | 0.9 (0.8-1.1) | -0.1 (-0.13--0.07) | 0.3 (0.2-0.6) | -0.28 (-0.44--0.13) | 18.5 (12.7-30.1) | -0.2 (-0.32--0.08) |  |
|  | Estonia | 1.1 (1-1.3) | -0.4 (-0.45--0.36) | 0.5 (0.3-0.7) | -1.86 (-2.1--1.62) | 25.3 (16.7-33.5) | -1.63 (-1.83--1.43) |  |
|  | Latvia | 1.3 (1.1-1.4) | -0.21 (-0.23--0.19) | 0.7 (0.4-0.9) | -0.99 (-1.23--0.75) | 33.3 (22.2-43.5) | -0.91 (-1.1--0.71) |  |
|  | Lithuania | 1.2 (1-1.3) | -0.21 (-0.24--0.17) | 0.6 (0.4-0.8) | -0.92 (-1.15--0.7) | 30.8 (21.9-38.9) | -0.85 (-1.05--0.66) |  |
|  | Republic of Moldova | 0.7 (0.6-0.8) | 0.19 (0.13-0.25) | 0.2 (0.2-0.3) | 0.05 (-0.21-0.3) | 12.7 (9.9-17.8) | 0.16 (-0.02-0.34) |  |
|  | Russian Federation | 0.9 (0.8-1.1) | -0.57 (-0.62--0.53) | 0.4 (0.3-0.9) | -1.67 (-1.97--1.37) | 22.3 (15.4-39.6) | -1.47 (-1.73--1.22) |  |
|  | Ukraine | 1.5 (1.3-1.7) | -0.46 (-0.56--0.36) | 0.8 (0.4-1.8) | -0.63 (-0.97--0.28) | 41.3 (26.1-83.4) | -0.5 (-0.77--0.23) |  |
|  | Central Europe | | | | | | |  |
|  | Albania | 3 (2.8-3.3) | -0.82 (-1.06--0.58) | 0.9 (0.5-1.7) | -2.88 (-3.34--2.41) | 41.6 (25.8-78.4) | -1.34 (-1.53--1.14) |  |
|  | Bosnia and Herzegovina | 1.1 (1-1.3) | -0.04 (-0.06--0.02) | 0.4 (0.3-0.7) | -0.77 (-0.84--0.69) | 21.9 (17.1-30.3) | -0.56 (-0.62--0.51) |  |
|  | Bulgaria | 1.9 (1.7-2.1) | -0.21 (-0.38--0.03) | 0.7 (0.5-1.2) | -1.12 (-1.74--0.5) | 33.1 (23.8-52.5) | -0.5 (-0.9--0.1) |  |
|  | Croatia | 1.2 (1-1.3) | 0.72 (0.63-0.81) | 0.6 (0.3-0.8) | 0.26 (0.17-0.36) | 26.5 (17.1-34.9) | 0.33 (0.25-0.41) |  |
|  | Czech Republic | 1.4 (1.2-1.5) | -0.43 (-0.48--0.37) | 0.6 (0.4-0.8) | -1.91 (-2.11--1.72) | 27.3 (21.4-36.5) | -1.59 (-1.73--1.45) |  |
|  | Hungary | 1.4 (1.3-1.6) | -0.84 (-1.12--0.56) | 0.6 (0.4-0.8) | -1.1 (-1.29--0.91) | 29 (22.8-37.8) | -1.1 (-1.17--1.03) |  |
|  | North Macedonia | 1.6 (1.4-1.8) | 0.65 (0.58-0.72) | 0.5 (0.4-0.7) | 0.17 (0.05-0.29) | 27 (21.1-34.4) | 0.21 (0.1-0.32) |  |
|  | Montenegro | 2 (1.9-2.2) | 0.19 (0.1-0.28) | 0.8 (0.5-1.2) | -0.18 (-0.32--0.03) | 38 (28.8-54.5) | -0.13 (-0.27-0) |  |
|  | Poland | 2.3 (2-2.6) | -0.29 (-0.32--0.27) | 0.9 (0.6-1.8) | -1.5 (-1.58--1.41) | 45.2 (31.8-77.3) | -1.16 (-1.23--1.09) |  |
|  | Romania | 0.8 (0.6-0.9) | -0.37 (-0.38--0.35) | 0.3 (0.2-0.5) | -2.82 (-3.04--2.61) | 15.2 (11.9-20.8) | -2.41 (-2.58--2.23) |  |
|  | Serbia | 1.9 (1.7-2.1) | 0.58 (0.5-0.67) | 0.8 (0.6-1.1) | -0.02 (-0.12-0.07) | 40.7 (32.2-50.2) | 0.08 (-0.01-0.17) |  |
|  | Slovakia | 1.2 (1-1.3) | 0.36 (0.29-0.44) | 0.5 (0.3-0.6) | 0.57 (0.2-0.94) | 22.8 (16.1-29.4) | 0.47 (0.22-0.72) |  |
|  | Slovenia | 1.7 (1.5-1.9) | 0.15 (0.08-0.21) | 0.7 (0.3-0.9) | -0.79 (-0.94--0.65) | 31.1 (20.9-40) | -0.54 (-0.64--0.45) |  |
|  | Central Asia | | | | | | |  |
|  | Armenia | 1.4 (1.2-1.6) | 0.83 (0.8-0.86) | 0.2 (0.2-0.3) | 1.03 (0.84-1.22) | 16 (11.9-19.9) | 0.87 (0.76-0.99) |  |
|  | Azerbaijan | 0.9 (0.8-1.1) | 0.33 (0.19-0.46) | 0.1 (0.1-0.2) | 0.2 (0.1-0.3) | 9.2 (7.1-11.5) | 0.11 (0.02-0.2) |  |
|  | Georgia | 1.1 (0.9-1.3) | 0.81 (0.76-0.87) | 0.2 (0.1-0.2) | 2.57 (2.18-2.96) | 12 (8.4-15.2) | 1.32 (1.2-1.44) |  |
|  | Kazakhstan | 2.8 (2.5-3.1) | 0.1 (-0.04-0.24) | 0.4 (0.3-0.6) | -0.22 (-0.32--0.11) | 24.7 (18-34.1) | 0.06 (-0.08-0.2) |  |
|  | Kyrgyzstan | 0.9 (0.7-1) | 0.09 (-0.08-0.26) | 0.1 (0.1-0.2) | -1.17 (-1.37--0.97) | 8.9 (6.8-11.2) | -0.61 (-0.72--0.5) |  |
|  | Mongolia | 1 (0.9-1.2) | 0.11 (-0.14-0.36) | 0.1 (0.1-0.2) | 2.15 (1.78-2.53) | 9.2 (6.8-12.1) | 0.91 (0.81-1) |  |
|  | Tajikistan | 0.8 (0.7-1) | 0.25 (0.1-0.41) | 0.1 (0.1-0.2) | 0.09 (-0.09-0.27) | 8.7 (6.8-10.9) | 0.04 (-0.1-0.19) |  |
|  | Turkmenistan | 1.6 (1.4-1.8) | 0.09 (0.07-0.1) | 0.3 (0.2-0.4) | 0.24 (-0.05-0.53) | 16.8 (13-21.3) | 0.33 (0.21-0.45) |  |
|  | Uzbekistan | 1 (0.9-1.2) | -0.76 (-0.8--0.71) | 0.1 (0.1-0.3) | -1.52 (-1.89--1.14) | 10.2 (7.5-14) | -0.73 (-0.82--0.63) |  |
|  | **Latin America and Caribbean** | | | | | | |  |
|  | Central Latin America | | | | | | |  |
|  | Colombia | 0.2 (0.2-0.3) | 0.45 (0.33-0.57) | 0.2 (0.1-0.2) | 0.73 (0.55-0.92) | 6.5 (4.7-8.7) | 0.76 (0.6-0.92) |  |
|  | Costa Rica | 0.3 (0.3-0.4) | 0.56 (0.39-0.74) | 0.2 (0.1-0.3) | 0.34 (0.14-0.53) | 8.5 (6.6-10.7) | 0.47 (0.33-0.62) |  |
|  | El Salvador | 0.3 (0.2-0.3) | 0.45 (0.43-0.47) | 0.1 (0.1-0.2) | 0.54 (0.44-0.64) | 5.6 (4.1-7.2) | 0.51 (0.43-0.58) |  |
|  | Guatemala | 0.3 (0.3-0.4) | 0.64 (0.61-0.68) | 0.2 (0.1-0.2) | 1.2 (1.01-1.4) | 7.2 (5.2-9.1) | 1.05 (0.92-1.18) |  |
|  | Honduras | 0.3 (0.2-0.3) | 0.36 (0.34-0.37) | 0.2 (0.1-0.3) | 1.32 (1.22-1.43) | 7.9 (5.1-12.4) | 1.14 (0.98-1.31) |  |
|  | Mexico | 0.5 (0.4-0.6) | 0.86 (0.76-0.96) | 0.3 (0.2-0.3) | 1.75 (1.58-1.93) | 12 (9.8-14.7) | 1.55 (1.4-1.7) |  |
|  | Nicaragua | 0.3 (0.2-0.3) | 0.41 (0.36-0.45) | 0.2 (0.1-0.2) | 0.86 (0.66-1.05) | 6.3 (5-7.8) | 0.58 (0.47-0.69) |  |
|  | Panama | 0.2 (0.2-0.3) | 0.25 (0.17-0.34) | 0.1 (0.1-0.2) | -0.12 (-0.34-0.09) | 6 (4.3-7.7) | -0.02 (-0.21-0.17) |  |
|  | Bolivarian Republic of Venezuela | 0.3 (0.2-0.3) | 0.47 (0.32-0.62) | 0.2 (0.1-0.3) | 0.51 (0.37-0.64) | 8.7 (6.4-11.4) | 0.57 (0.45-0.69) |  |
|  | Andean Latin America | | | | | | |  |
|  | Bolivia | 0.4 (0.3-0.5) | 0.4 (0.37-0.43) | 0.2 (0.2-0.3) | 0.3 (0.26-0.34) | 8.7 (6.7-11.3) | 0.21 (0.17-0.25) |  |
|  | Ecuador | 0.3 (0.2-0.3) | 0.62 (0.52-0.71) | 0.1 (0.1-0.2) | 0.68 (0.38-0.98) | 5.9 (4.6-7.4) | 0.6 (0.4-0.81) |  |
|  | Peru | 0.3 (0.3-0.4) | 0.7 (0.64-0.76) | 0.1 (0.1-0.2) | 0.05 (-0.12-0.21) | 5.5 (4.1-7.3) | 0.22 (0.11-0.33) |  |
|  | Caribbean | | | | | | |  |
|  | Antigua and Barbuda | 0.8 (0.7-0.9) | 0.79 (0.59-0.99) | 0.4 (0.2-0.5) | 0.76 (0.67-0.85) | 16.9 (12.7-20.8) | 0.65 (0.59-0.71) |  |
|  | The Bahamas | 0.6 (0.5-0.7) | 0.39 (0.36-0.43) | 0.3 (0.2-0.4) | 0.68 (0.58-0.79) | 14.8 (11.5-18.5) | 0.67 (0.59-0.74) |  |
|  | Barbados | 0.7 (0.6-0.8) | 0.78 (0.6-0.96) | 0.4 (0.3-0.6) | 1 (0.87-1.12) | 20.1 (15.2-25) | 0.88 (0.75-1.01) |  |
|  | Belize | 0.4 (0.3-0.5) | 0.35 (0.3-0.4) | 0.1 (0.1-0.2) | 0.79 (0.65-0.94) | 7.4 (6-10.1) | 0.79 (0.69-0.88) |  |
|  | Bermuda | 0.7 (0.5-0.8) | 0.25 (0.22-0.27) | 0.2 (0.1-0.3) | -0.76 (-0.9--0.62) | 11.2 (8.6-15) | -0.34 (-0.42--0.25) |  |
|  | Cuba | 0.6 (0.5-0.7) | 0.42 (0.34-0.5) | 0.3 (0.2-0.4) | 0.42 (0.31-0.53) | 13.6 (9.7-17.1) | 0.27 (0.18-0.35) |  |
|  | Dominica | 0.4 (0.3-0.4) | 0.28 (0.26-0.3) | 0.1 (0.1-0.2) | 0.29 (0.25-0.34) | 7.5 (5.9-9.5) | 0.25 (0.22-0.28) |  |
|  | Dominican Republic | 0.4 (0.3-0.5) | 0.27 (0.2-0.34) | 0.2 (0.1-0.2) | 1.59 (1.43-1.75) | 7.6 (5.6-10.3) | 1.18 (1.07-1.29) |  |
|  | Grenada | 0.5 (0.4-0.6) | 0.39 (0.23-0.54) | 0.3 (0.3-0.5) | 0.19 (-0.04-0.41) | 14.6 (11.9-20.2) | 0.1 (-0.1-0.3) |  |
|  | Guyana | 0.3 (0.2-0.3) | 0.29 (0.29-0.3) | 0.1 (0.1-0.2) | 0.59 (0.52-0.67) | 6.7 (5-9.3) | 0.72 (0.64-0.79) |  |
|  | Haiti | 0.5 (0.4-0.6) | 0.35 (0.34-0.35) | 0.3 (0.2-0.5) | 0.33 (0.23-0.43) | 12.5 (8.3-20.9) | 0.42 (0.34-0.5) |  |
|  | Jamaica | 0.4 (0.3-0.5) | 0.29 (0.25-0.33) | 0.1 (0.1-0.2) | 0.61 (0.45-0.76) | 7.1 (5.5-8.8) | 0.56 (0.45-0.68) |  |
|  | Puerto Rico | 0.5 (0.4-0.6) | 0.44 (0.34-0.53) | 0.3 (0.2-0.4) | 0.85 (0.6-1.11) | 13.8 (9.2-18.2) | 0.83 (0.62-1.03) |  |
|  | Saint Lucia | 0.4 (0.3-0.4) | 0.29 (0.25-0.32) | 0.2 (0.1-0.3) | 0.31 (0.16-0.46) | 9.1 (7.5-11.8) | 0.45 (0.35-0.56) |  |
|  | Saint Vincent and the Grenadines | 0.4 (0.3-0.4) | 0.39 (0.37-0.42) | 0.2 (0.1-0.2) | 0.29 (0.19-0.4) | 8.8 (6.9-10.6) | 0.32 (0.24-0.39) |  |
|  | Suriname | 0.3 (0.2-0.3) | 0.38 (0.34-0.41) | 0.2 (0.1-0.2) | 0.52 (0.35-0.69) | 7.3 (5.8-9.4) | 0.55 (0.41-0.69) |  |
|  | Trinidad and Tobago | 0.3 (0.3-0.4) | 0.37 (0.35-0.4) | 0.2 (0.1-0.2) | 0.92 (0.76-1.09) | 8.2 (5-11.9) | 1.13 (0.97-1.29) |  |
|  | United States Virgin Islands | 0.5 (0.4-0.6) | 0.38 (0.3-0.46) | 0.2 (0.2-0.3) | 0.14 (0.06-0.21) | 10.4 (8.3-13.5) | 0.08 (0.03-0.12) |  |
|  | Tropical Latin America | | | | | | |  |
|  | Brazil | 0.8 (0.7-0.9) | 0.25 (0.16-0.34) | 0.2 (0.1-0.2) | -0.08 (-0.31-0.15) | 10.1 (8.1-12.8) | 0.29 (0.19-0.38) |  |
|  | Paraguay | 0.6 (0.5-0.7) | 0.15 (0.03-0.28) | 0.1 (0.1-0.2) | 1.21 (1.12-1.3) | 8.1 (5.5-10.5) | 0.71 (0.64-0.79) |  |
|  | **Southeast Asia, east Asia, and Oceania** | | | | | | |  |
|  | East Asia | | | | | | |  |
|  | China | 0.2 (0.1-0.2) | -0.17 (-0.27--0.07) | 0.1 (0.1-0.1) | -1.48 (-1.63--1.33) | 3.7 (3-4.8) | -1.4 (-1.58--1.21) |  |
|  | Democratic People's Republic of Korea | 0.3 (0.2-0.3) | -0.15 (-0.21--0.08) | 0.1 (0.1-0.2) | -0.52 (-0.55--0.49) | 6.2 (4.5-8.8) | -0.52 (-0.57--0.47) |  |
|  | Taiwan (Province of China) | 0.3 (0.2-0.3) | 1.46 (1.14-1.78) | 0.1 (0.1-0.1) | -0.36 (-0.42--0.31) | 5.2 (4.1-6.9) | 0.42 (0.3-0.55) |  |
|  | Southeast Asia | | | | | | |  |
|  | Cambodia | 0.2 (0.1-0.2) | -0.13 (-0.13--0.12) | 0.1 (0.1-0.2) | -0.16 (-0.24--0.08) | 5 (3.8-7.1) | -0.3 (-0.37--0.23) |  |
|  | Indonesia | 0.2 (0.1-0.2) | -0.02 (-0.04-0) | 0.1 (0.1-0.2) | 0.25 (0.17-0.33) | 4 (2.8-5.9) | -0.05 (-0.13-0.04) |  |
|  | Lao People's Democratic Republic | 0.2 (0.2-0.3) | -0.14 (-0.17--0.1) | 0.1 (0.1-0.2) | -0.94 (-1.03--0.85) | 5.1 (3.8-7.3) | -0.93 (-1.01--0.85) |  |
|  | Malaysia | 0.1 (0.1-0.1) | -0.06 (-0.09--0.03) | 0.1 (0.1-0.1) | 0.01 (-0.07-0.09) | 3.7 (2.8-4.8) | -0.01 (-0.08-0.05) |  |
|  | Maldives | 0.1 (0.1-0.1) | -0.33 (-0.37--0.29) | 0.1 (0-0.1) | -1.41 (-1.56--1.25) | 2.9 (1.7-4.8) | -1.37 (-1.51--1.23) |  |
|  | Mauritius | 0.2 (0.2-0.3) | 0.01 (-0.04-0.06) | 0.1 (0.1-0.2) | -0.17 (-0.33--0.01) | 4.5 (3.6-6) | -0.11 (-0.24-0.01) |  |
|  | Myanmar | 0.3 (0.2-0.3) | -0.12 (-0.13--0.11) | 0.1 (0.1-0.2) | -0.75 (-0.84--0.65) | 5.6 (4.2-8) | -0.83 (-0.92--0.73) |  |
|  | Philippines | 0.2 (0.2-0.2) | 0.07 (0.04-0.1) | 0.1 (0.1-0.2) | -0.77 (-0.97--0.56) | 4.8 (3.6-7.4) | -0.69 (-0.86--0.52) |  |
|  | Sri Lanka | 0.2 (0.1-0.2) | -0.09 (-0.17--0.02) | 0.1 (0.1-0.3) | -1.99 (-2.3--1.68) | 5 (2.9-9.9) | -3.12 (-3.63--2.6) |  |
|  | Seychelles | 0.2 (0.2-0.2) | 0.01 (-0.13-0.16) | 0.2 (0.1-0.3) | 0.4 (0.17-0.62) | 8.7 (4.4-13.1) | 0.45 (0.2-0.69) |  |
|  | Thailand | 0.2 (0.1-0.2) | -0.13 (-0.14--0.12) | 0.1 (0.1-0.1) | -1.55 (-1.86--1.24) | 4.1 (3-5.8) | -2 (-2.44--1.56) |  |
|  | Timor-Leste | 0.2 (0.1-0.2) | -0.01 (-0.03-0.01) | 0.1 (0.1-0.1) | 0.5 (0.22-0.78) | 4 (2.6-5.5) | 0.28 (0-0.56) |  |
|  | Vietnam | 0.2 (0.2-0.2) | -0.03 (-0.04--0.02) | 0.1 (0.1-0.2) | 0.28 (0.22-0.33) | 5.4 (4-7.4) | 0.2 (0.15-0.26) |  |
|  | Oceania | | | | | | |  |
|  | American Samoa | 0.2 (0.1-0.2) | -0.01 (-0.03-0.02) | 0.1 (0.1-0.1) | -0.4 (-0.52--0.28) | 3.7 (3-4.7) | -0.31 (-0.41--0.2) |  |
|  | Federated States of Micronesia | 0.1 (0.1-0.2) | -0.12 (-0.16--0.07) | 0.1 (0.1-0.2) | -0.61 (-0.76--0.47) | 5.1 (3-7.5) | -0.58 (-0.68--0.47) |  |
|  | Fiji | 0.2 (0.1-0.2) | -0.04 (-0.07-0) | 0.1 (0.1-0.1) | -0.35 (-0.42--0.29) | 4.1 (3.2-5.3) | -0.31 (-0.37--0.24) |  |
|  | Guam | 0.1 (0.1-0.2) | -0.03 (-0.05--0.01) | 0.1 (0.1-0.1) | -0.92 (-1.07--0.77) | 3.1 (2.6-3.9) | -0.47 (-0.55--0.39) |  |
|  | Kiribati | 0.1 (0.1-0.2) | -0.12 (-0.25-0.01) | 0.2 (0.1-0.3) | -0.66 (-0.75--0.57) | 7.1 (4.8-11.7) | -0.64 (-0.73--0.56) |  |
|  | Marshall Islands | 0.2 (0.1-0.2) | -0.05 (-0.11-0) | 0.1 (0.1-0.2) | -0.46 (-0.49--0.43) | 5.3 (3.9-7.5) | -0.35 (-0.38--0.32) |  |
|  | Northem Mariana lslands | 0.2 (0.1-0.2) | -0.06 (-0.09--0.03) | 0.1 (0.1-0.1) | -0.47 (-0.5--0.45) | 4.2 (3.3-5.2) | -0.45 (-0.48--0.43) |  |
|  | Papua New Guinea | 0.1 (0.1-0.2) | -0.05 (-0.07--0.03) | 0.1 (0-0.1) | -0.21 (-0.26--0.16) | 3.1 (2-4.5) | -0.21 (-0.26--0.16) |  |
|  | Samoa | 0.2 (0.1-0.2) | -0.08 (-0.08--0.07) | 0.1 (0.1-0.2) | -0.86 (-1.01--0.72) | 4.5 (3.4-6) | -0.72 (-0.83--0.61) |  |
|  | Solomon Islands | 0.2 (0.1-0.2) | -0.1 (-0.14--0.06) | 0.1 (0.1-0.2) | -0.29 (-0.36--0.21) | 5.8 (3.7-9.9) | -0.23 (-0.29--0.17) |  |
|  | Tonga | 0.2 (0.2-0.2) | -0.04 (-0.08-0) | 0.1 (0.1-0.1) | -0.32 (-0.42--0.23) | 4.3 (3.1-5.7) | -0.28 (-0.36--0.21) |  |
|  | Vanuatu | 0.2 (0.1-0.2) | -0.05 (-0.07--0.03) | 0.1 (0.1-0.2) | -0.34 (-0.53--0.15) | 5.1 (3.5-7.3) | -0.26 (-0.44--0.09) |  |
|  | Cook Islands | 0.2 (0.2-0.2) | -0.01 (-0.03-0.02) | 0.1 (0.1-0.1) | -1.18 (-1.4--0.97) | 4 (3-5.2) | -0.95 (-1.15--0.75) |  |
|  | **North Africa and Middle East** | | | | | | |  |
|  | North Africa and Middle East | | | | | | |  |
|  | Afghanistan | 1.6 (1.4-1.9) | 0.46 (0.4-0.52) | 0.4 (0.2-0.9) | 0.24 (0.1-0.38) | 27.1 (16.8-48.7) | 0.33 (0.25-0.42) |  |
|  | Algeria | 1.5 (1.3-1.8) | 0.64 (0.62-0.66) | 0.3 (0.1-0.4) | 0.05 (-0.02-0.11) | 20.1 (13.6-26.4) | 0.38 (0.33-0.43) |  |
|  | Bahrain | 1.2 (1-1.5) | 0.77 (0.73-0.81) | 0.1 (0.1-0.2) | -0.23 (-0.35--0.11) | 12.5 (9.1-16.2) | 0.35 (0.3-0.39) |  |
|  | Egypt | 0.8 (0.7-1) | 0.56 (0.52-0.61) | 0.2 (0.1-0.4) | 0.2 (0.07-0.33) | 15.3 (9.6-32.4) | 0.21 (0.08-0.34) |  |
|  | Iran | 2 (1.7-2.2) | 0.37 (0.17-0.57) | 0.5 (0.4-0.7) | 0.24 (0.16-0.31) | 29.2 (23.7-37.4) | 0.36 (0.24-0.48) |  |
|  | Iraq | 1.3 (1.1-1.6) | 0.47 (0.37-0.58) | 0.1 (0.1-0.2) | -0.06 (-0.17-0.04) | 14.3 (10.6-18.6) | 0.32 (0.29-0.35) |  |
|  | Jordan | 1.6 (1.3-1.9) | -0.25 (-0.38--0.13) | 0.2 (0.1-0.2) | -0.73 (-0.87--0.59) | 19 (13.6-23.9) | -0.43 (-0.57--0.29) |  |
|  | Kuwait | 1.8 (1.5-2.1) | 1.46 (1.34-1.59) | 0.1 (0.1-0.2) | 0.23 (0.01-0.45) | 17.3 (12.8-22.6) | 1.25 (1.07-1.42) |  |
|  | Lebanon | 1.8 (1.5-2.2) | 0.85 (0.81-0.89) | 0.3 (0.1-0.4) | 0.27 (0.18-0.36) | 23.5 (16.4-30.3) | 0.69 (0.66-0.72) |  |
|  | Libya | 1.5 (1.3-1.7) | 0.78 (0.71-0.86) | 0.3 (0.2-0.5) | 1.22 (1.09-1.34) | 21.8 (14.9-30.2) | 1.07 (0.97-1.18) |  |
|  | Morocco | 1.5 (1.2-1.8) | 0.63 (0.6-0.65) | 0.3 (0.1-0.4) | 0.99 (0.92-1.05) | 20.2 (14.1-27) | 0.79 (0.74-0.85) |  |
|  | Oman | 1.2 (1-1.4) | 0.76 (0.6-0.92) | 0.3 (0.1-0.4) | 0.66 (0.23-1.09) | 17.1 (11.6-23.2) | 0.62 (0.29-0.96) |  |
|  | Palestine | 1.5 (1.2-1.7) | 0.52 (0.5-0.54) | 0.2 (0.2-0.4) | 0.03 (-0.05-0.11) | 19.5 (15.3-24.3) | 0.27 (0.22-0.32) |  |
|  | Qatar | 1.8 (1.6-2) | 0.51 (0.16-0.86) | 0.1 (0.1-0.1) | -0.69 (-0.84--0.55) | 16.8 (12.3-21.8) | 0.32 (0.03-0.6) |  |
|  | Saudi Arabia | 1 (0.8-1.2) | 0.72 (0.68-0.75) | 0.1 (0.1-0.2) | 0.39 (0.26-0.52) | 11.7 (8.7-15) | 0.65 (0.6-0.71) |  |
|  | Sudan | 0.9 (0.7-1) | 0.53 (0.52-0.54) | 0.2 (0.1-0.3) | 0.63 (0.61-0.65) | 14 (8.9-19.2) | 0.65 (0.63-0.66) |  |
|  | Syrian Arab Republic | 1.5 (1.2-1.8) | 0.68 (0.58-0.77) | 0.1 (0.1-0.2) | -0.03 (-0.15-0.1) | 15.2 (11.3-19.6) | 0.47 (0.4-0.55) |  |
|  | Tunisia | 1.7 (1.4-2.1) | 0.73 (0.72-0.74) | 0.3 (0.1-0.4) | 0.72 (0.6-0.85) | 22.2 (15.5-29.2) | 0.77 (0.7-0.84) |  |
|  | Turkey | 1.7 (1.6-1.8) | -0.09 (-0.09--0.08) | 0.2 (0.2-0.3) | -1.16 (-1.26--1.05) | 21 (16.3-27.6) | -0.56 (-0.61--0.5) |  |
|  | United Arab Emirates | 1.1 (0.9-1.2) | 0.25 (0.19-0.31) | 0.4 (0.2-0.7) | 0.63 (0.35-0.9) | 20.6 (12.3-32.5) | 0.59 (0.5-0.69) |  |
|  | Yemen | 0.9 (0.7-1) | 0.58 (0.57-0.59) | 0.2 (0.1-0.3) | 1.03 (0.94-1.11) | 14.1 (9.2-19.6) | 0.91 (0.85-0.97) |  |
|  | **South Asia** | | | | | | |  |
|  | South Asia | | | | | | |  |
|  | Bangladesh | 0.4 (0.3-0.4) | 0.08 (0.04-0.11) | 0.2 (0.1-0.2) | -0.4 (-0.47--0.33) | 7.3 (5-9.7) | -0.21 (-0.28--0.15) |  |
|  | Bhutan | 0.4 (0.3-0.5) | 0.12 (0.05-0.18) | 0.2 (0.1-0.3) | 0.29 (0.25-0.33) | 8.2 (5.3-11.3) | 0.14 (0.09-0.19) |  |
|  | India | 0.4 (0.3-0.5) | 0.36 (0.32-0.39) | 0.2 (0.1-0.2) | 0.19 (0.1-0.28) | 8.4 (6.9-10.4) | 0.3 (0.23-0.37) |  |
|  | Nepal | 0.4 (0.4-0.5) | 0.23 (0.16-0.31) | 0.2 (0.1-0.3) | 0.48 (0.28-0.67) | 8.9 (6.1-11.9) | 0.33 (0.16-0.49) |  |
|  | Pakistan | 0.5 (0.4-0.6) | 0.24 (0.17-0.31) | 0.2 (0.2-0.4) | 0.6 (0.43-0.77) | 10.9 (8.3-15.5) | 0.57 (0.45-0.69) |  |
|  | **Sub-Saharan Africa** | | | | | | |  |
|  | Southern sub-Saharan Africa | | | | | | |  |
|  | Botswana | 0.3 (0.3-0.4) | 0.07 (0-0.14) | 0.2 (0.1-0.2) | 0.26 (-0.08-0.61) | 7 (4.9-9.7) | 0.3 (0.05-0.55) |  |
|  | Lesotho | 0.4 (0.3-0.5) | 0.14 (0.03-0.25) | 0.2 (0.1-0.3) | 2.1 (1.86-2.34) | 8.1 (5.7-11.4) | 1.6 (1.44-1.76) |  |
|  | Namibia | 0.3 (0.3-0.4) | 0.01 (-0.06-0.08) | 0.1 (0.1-0.2) | 0.24 (0.03-0.46) | 6.2 (4.6-8.2) | 0.28 (0.13-0.43) |  |
|  | South Africa | 0.4 (0.3-0.5) | 0.06 (-0.05-0.17) | 0.1 (0.1-0.2) | -0.14 (-0.34-0.06) | 6.5 (5.4-7.8) | -0.21 (-0.3--0.13) |  |
|  | Eswatini | 0.4 (0.3-0.5) | 0.1 (0-0.2) | 0.2 (0.1-0.2) | 0.89 (0.52-1.26) | 7.4 (5.2-10.2) | 0.78 (0.54-1.03) |  |
|  | Zimbabwe | 0.3 (0.3-0.4) | 0.2 (0.15-0.26) | 0.2 (0.1-0.2) | 1.24 (1.04-1.44) | 7.2 (5-10) | 1.09 (0.95-1.24) |  |
|  | Western sub-Saharan Africa | | | | | | |  |
|  | Benin | 0.4 (0.3-0.4) | 0.08 (0.07-0.1) | 0.2 (0.1-0.3) | 0.24 (0.18-0.3) | 8.6 (6.3-12) | 0.36 (0.31-0.41) |  |
|  | Burkina Faso | 0.4 (0.3-0.5) | 0.07 (0.03-0.1) | 0.2 (0.1-0.3) | 0.35 (0.17-0.53) | 9.2 (6.3-13.1) | 0.48 (0.34-0.62) |  |
|  | Cameroon | 0.3 (0.3-0.4) | 0.11 (0.08-0.14) | 0.2 (0.1-0.4) | 0.5 (0.43-0.56) | 9.4 (6.6-14.5) | 0.59 (0.53-0.64) |  |
|  | Cape Verde | 0.4 (0.3-0.5) | 0.08 (0.03-0.13) | 0.2 (0.1-0.3) | 0.85 (0.77-0.92) | 9.1 (6.7-11.9) | 0.65 (0.61-0.69) |  |
|  | Chad | 0.4 (0.3-0.5) | 0.11 (0.05-0.16) | 0.2 (0.1-0.3) | 0.51 (0.44-0.59) | 9.2 (6.5-13.3) | 0.54 (0.49-0.59) |  |
|  | Côte d'Ivoire | 0.3 (0.3-0.4) | 0.16 (0.14-0.19) | 0.2 (0.1-0.3) | 0.09 (0-0.17) | 8.1 (5.8-11.4) | 0.24 (0.17-0.31) |  |
|  | The Gambia | 0.4 (0.3-0.5) | 0.3 (0.29-0.31) | 0.2 (0.2-0.4) | 1.06 (0.87-1.25) | 10 (7.4-14) | 1.04 (0.88-1.2) |  |
|  | Ghana | 1 (0.9-1.1) | 1.28 (1.11-1.45) | 0.6 (0.3-0.9) | 2.09 (1.77-2.42) | 25.2 (16.3-34.4) | 1.94 (1.68-2.21) |  |
|  | Guinea | 0.4 (0.3-0.5) | 0.22 (0.2-0.24) | 0.2 (0.2-0.3) | 0.76 (0.72-0.81) | 9.3 (7-12.9) | 0.82 (0.78-0.86) |  |
|  | Guinea-Bissau | 0.4 (0.3-0.5) | 0.07 (0.06-0.09) | 0.3 (0.2-0.4) | 0.38 (0.33-0.44) | 11.7 (8.3-17.3) | 0.46 (0.4-0.51) |  |
|  | Liberia | 0.3 (0.2-0.4) | 0.07 (0.04-0.1) | 0.2 (0.1-0.3) | 0.24 (0.1-0.38) | 7.6 (5.7-10.4) | 0.38 (0.26-0.5) |  |
|  | Mali | 0.4 (0.4-0.5) | 0.06 (0.01-0.12) | 0.2 (0.1-0.4) | 0.04 (-0.03-0.11) | 10 (7.1-14.3) | 0.13 (0.07-0.2) |  |
|  | Mauritania | 0.5 (0.4-0.6) | 0.09 (0.07-0.11) | 0.2 (0.2-0.4) | -0.4 (-0.5--0.29) | 10.3 (7.4-14.6) | -0.12 (-0.21--0.03) |  |
|  | Niger | 0.4 (0.4-0.5) | 0.07 (0.01-0.13) | 0.2 (0.1-0.3) | 0.11 (0.02-0.19) | 9.1 (5.6-13.4) | 0.22 (0.15-0.29) |  |
|  | Nigeria | 0.3 (0.3-0.4) | 0.29 (0.24-0.35) | 0.2 (0.1-0.3) | 1.14 (0.98-1.29) | 7.9 (5.7-11.2) | 1.04 (0.92-1.17) |  |
|  | São Tomé and Príncipe | 0.2 (0.2-0.3) | 0.18 (0.16-0.2) | 0.2 (0.1-0.3) | 1.13 (1.02-1.23) | 6.8 (4.7-10.2) | 0.9 (0.79-1.01) |  |
|  | Senegal | 0.4 (0.3-0.5) | 0.16 (0.14-0.18) | 0.2 (0.2-0.3) | 0.65 (0.47-0.83) | 9.9 (7.3-13.6) | 0.67 (0.51-0.82) |  |
|  | Sierra Leone | 0.3 (0.3-0.4) | 0.16 (0.13-0.18) | 0.2 (0.1-0.3) | 0.84 (0.76-0.91) | 8 (6-11) | 0.9 (0.83-0.97) |  |
|  | Togo | 0.4 (0.3-0.4) | 0.14 (0.13-0.16) | 0.2 (0.2-0.3) | 0.41 (0.36-0.46) | 9.3 (6.8-13) | 0.52 (0.47-0.56) |  |
|  | Eastern sub-Saharan Africa | | | | | | |  |
|  | Burundi | 0.2 (0.2-0.3) | -0.08 (-0.14--0.01) | 0.1 (0-0.1) | -0.49 (-0.58--0.4) | 4.3 (2.6-6) | -0.4 (-0.48--0.33) |  |
|  | Comoros | 0.3 (0.2-0.3) | 0 (-0.04-0.04) | 0.1 (0.1-0.2) | 0.28 (0.08-0.47) | 5.7 (3.8-8) | 0.24 (0.06-0.43) |  |
|  | Djibouti | 0.3 (0.2-0.3) | 0.07 (0.02-0.12) | 0.1 (0.1-0.2) | 0.57 (0.51-0.63) | 5.6 (3.7-8.2) | 0.47 (0.42-0.52) |  |
|  | Eritrea | 0.3 (0.3-0.4) | 0.03 (-0.04-0.09) | 0.2 (0.1-0.2) | 0.64 (0.5-0.78) | 6.9 (4.7-9.8) | 0.52 (0.43-0.6) |  |
|  | Ethiopia | 0.3 (0.2-0.3) | -0.11 (-0.22-0) | 0.1 (0-0.2) | -1.07 (-1.22--0.91) | 4.8 (2.8-6.7) | -0.92 (-1.06--0.78) |  |
|  | Kenya | 0.2 (0.2-0.3) | 0.12 (0.07-0.16) | 0.1 (0.1-0.2) | 1.24 (1.12-1.36) | 4.7 (3.5-6.8) | 0.99 (0.89-1.09) |  |
|  | Madagascar | 0.4 (0.3-0.4) | 0.04 (-0.06-0.15) | 0.1 (0.1-0.2) | -0.03 (-0.07-0.02) | 6 (4.3-8.4) | -0.03 (-0.08-0.02) |  |
|  | Malawi | 0.3 (0.2-0.4) | 0.07 (-0.02-0.15) | 0.1 (0.1-0.2) | 0.15 (0.09-0.21) | 5.5 (3.4-7.8) | 0.12 (0.06-0.18) |  |
|  | Mozambique | 0.4 (0.3-0.4) | 0.08 (-0.01-0.17) | 0.2 (0.1-0.2) | 1.03 (0.9-1.17) | 6.9 (4.1-10.2) | 0.85 (0.73-0.98) |  |
|  | Rwanda | 0.2 (0.2-0.2) | -0.07 (-0.09--0.05) | 0.1 (0.1-0.2) | -0.86 (-1.1--0.62) | 4.5 (3-6.2) | -0.71 (-0.9--0.52) |  |
|  | Somalia | 0.2 (0.2-0.3) | 0.04 (-0.03-0.11) | 0.1 (0-0.2) | 0.19 (0.15-0.23) | 4.6 (2.2-7.3) | 0.16 (0.13-0.19) |  |
|  | South Sudan | 0.2 (0.2-0.3) | 0.17 (0.1-0.24) | 0.1 (0-0.1) | -0.01 (-0.11-0.08) | 4.2 (2.6-5.9) | 0.07 (-0.03-0.17) |  |
|  | United Republic of Tanzania | 0.2 (0.2-0.3) | 0.06 (0-0.12) | 0.1 (0.1-0.2) | 0.4 (0.3-0.49) | 4.9 (3.2-6.7) | 0.36 (0.27-0.44) |  |
|  | Uganda | 0.2 (0.2-0.2) | 0.08 (0.02-0.14) | 0.1 (0.1-0.1) | 0.59 (0.5-0.69) | 4 (2.6-5.7) | 0.51 (0.42-0.61) |  |
|  | Zambia | 0.3 (0.2-0.4) | -0.03 (-0.07-0.01) | 0.1 (0.1-0.2) | 0.26 (0.2-0.33) | 6.6 (4.9-9.7) | 0.26 (0.21-0.3) |  |
|  | Central sub-Saharan Africa | | | | | | |  |
|  | Angola | 0.3 (0.2-0.3) | 0.09 (0.03-0.15) | 0.1 (0.1-0.2) | 0.13 (0.07-0.19) | 5.9 (4.2-8.3) | 0.15 (0.09-0.21) |  |
|  | Central African Republic | 0.2 (0.2-0.3) | 0.1 (0.05-0.16) | 0.1 (0.1-0.2) | -0.18 (-0.23--0.13) | 5.4 (3.5-8.8) | -0.12 (-0.15--0.08) |  |
|  | Congo | 0.2 (0.2-0.2) | 0.07 (0.06-0.08) | 0.1 (0.1-0.2) | -0.34 (-0.48--0.2) | 5.3 (3.5-9) | -0.26 (-0.38--0.15) |  |
|  | Democratic Republic of the Congo | 0.2 (0.2-0.2) | 0.07 (0.03-0.12) | 0.1 (0.1-0.2) | 0.06 (-0.04-0.15) | 4.6 (3-6.5) | 0.08 (-0.01-0.16) |  |
|  | Equatorial Guinea | 0.2 (0.2-0.2) | -0.13 (-0.14--0.11) | 0.1 (0.1-0.2) | 0.43 (0.26-0.59) | 4.8 (2.8-8.3) | 0.29 (0.16-0.41) |  |
|  | Gabon | 0.2 (0.2-0.2) | 0.2 (0.18-0.22) | 0.1 (0.1-0.2) | 0.08 (0-0.17) | 5.3 (3.5-8.8) | 0.11 (0.03-0.18) |  |
|  | **Supplemental table 1: Incidence, deaths, and DALYs for multiple sclerosis in 2019, and EAPC of ASR by countries.**  DALYs=disability-adjusted life years. ASR= age-standardized rates. EAPC= estimated annual percentage change. | | | | | | |  |

|  |  | **1990** | | **2019** | | **1990~2019** |  |
| --- | --- | --- | --- | --- | --- | --- | --- |
|  |  | **Number**  **No. (95 UI%)** | **ASR per 100，000**  **No. (95 UI%)** | **Number**  **No. (95 UI%)** | **ASR per 100，000**  **No. (95 UI%)** | **EAPC**  **No. ×100% (95 CI%)** |  |
|  | **Total neurological disorders** |  |  |  |  |  |  |
|  | Incidence | 539884532  (481388440.5-598081394.1) | 10266.1  (9263.8-11327.1) | 805178458.1  (725838130.6-888847391.9) | 10259.5  (9223.2-11324.2) | -0.01  (-0.02-0.01) |  |
|  | Deaths | 873338.9  (450771.2-1848823.4) | 30.3  (13.7-67.7) | 2221323.4  (1027903.2-4759829) | 30.7  (13.8-66.3) | 0.06  (0.03-0.08) |  |
|  | DALYs | 57515331.1  (31943598.4-93752878.9) | 1264.2  (740.3-2040.8) | 97724411.7  (55942819.3-159416792.6) | 1253.6  (719.7-2039.8) | -0.04  (-0.05--0.04) |  |
|  | **Multiple sclerosis** |  |  |  |  |  |  |
|  | Incidence | 41854  (36306.1-47444.9) | 0.8  (0.7-0.9) | 59345.4  (51817.8-66942.6) | 0.7  (0.6-0.8) | -0.19  (-0.24--0.13) |  |
|  | Deaths | 13356  (11903.5-17571.1) | 0.3  (0.3-0.4) | 22439  (20226-27791.5) | 0.3  (0.2-0.3) | -0.62  (-0.67--0.56) |  |
|  | DALYs | 726065.6  (621892.3-867796) | 16.1  (13.8-19.3) | 1159831.8  (1001179.9-1381870.2) | 14  (12-16.6) | -0.56  (-0.6--0.52) |  |
|  | **Alzheimer's disease and other dementias** |  |  |  |  |  |  |
|  | Incidence | 2920984.8  (2485352.5-3372858.7) | 93.6  (80.1-106.7) | 7236385  (6217238.9-8232671.5) | 95  (81.6-107.9) | 0.06  (0.03-0.08) |  |
|  | Deaths | 560934.8  (135270.4-1546915.5) | 22.2  (5.5-60) | 1623275.9  (407465.3-4205718.7) | 22.9  (5.8-59.2) | 0.13  (0.1-0.15) |  |
|  | DALYs | 9663312  (4233954.9-21374778.7) | 326.7  (143.3-731) | 25276988.9  (11204523-54558243.1) | 338.6  (151-731.3) | 0.15  (0.13-0.16) |  |
|  | **Idiopathic epilepsy** |  |  |  |  |  |  |
|  | Incidence | 1859516.8  (1281783-2526087) | 33.2  (23.4-44.7) | 2898222.2  (2098717.7-3823375.6) | 38.8  (28-51.3) | 0.49  (0.46-0.51) |  |
|  | Deaths | 100054.1  (81176.2-112226.4) | 1.9  (1.6-2.1) | 114010.9  (100177.7-129928.1) | 1.5  (1.3-1.7) | -1.11  (-1.17--1.04) |  |
|  | DALYs | 11285622.5  (8614047.6-14136603.8) | 204.3  (157.6-254.1) | 13077624.5  (9986729.6-16734085.8) | 170.6  (130.4-218.3) | -0.78  (-0.82--0.73) |  |
|  | **Parkinson's disease** |  |  |  |  |  |  |
|  | Incidence | 416474.3  (362669.3-473209.9) | 11.2  (9.9-12.7) | 1081722.7  (953265-1211202.2) | 13.4  (11.8-15) | 0.61  (0.58-0.65) |  |
|  | Deaths | 147237.6  (137334.6-158063) | 4.6  (4.3-4.9) | 362906.9  (326854.8-388199.8) | 4.8  (4.3-5.1) | 0.15  (0.09-0.22) |  |
|  | DALYs | 2749553.6  (2541530.2-2992889.4) | 78.1  (72.3-84.7) | 6292615.6  (5769209.8-6827206.9) | 80  (73.3-86.6) | 0.1  (0.04-0.16) |  |
|  | **Motor neuron disease** |  |  |  |  |  |  |
|  | Incidence | 35589.2  (31621.3-40068) | 0.8  (0.7-0.9) | 63700  (57295.9-71343.3) | 0.8  (0.7-0.9) | 0.03  (-0.03-0.09) |  |
|  | Deaths | 17653.2  (17010.7-18269.7) | 0.4  (0.4-0.4) | 39081.2  (36566.7-41129.6) | 0.5  (0.4-0.5) | 0.41  (0.35-0.46) |  |
|  | DALYs | 624364.4  (594254.2-665295.3) | 13.3  (12.7-13.9) | 1034606.6  (979910.9-1085401.1) | 12.7  (12-13.3) | -0.24  (-0.28--0.19) |  |
|  | **Headache disorders** |  |  |  |  |  |  |
|  | Incidence | 534610112.9  (476171975.6 - 592708956.9) | 10126.5  (9123.5 - 11170.4) | 793839082.7  (714299832.4 - 877018808.2) | 10110.7  (9070.7 - 11167.1) | -0.01  (-0.02 - 0) |  |
|  | DALYs | 29741448.5  (6040896.4 - 64204326.5) | 575.2  (124.6 - 1225.3) | 46619354.8  (9772903.2 - 100161725.6) | 581.8  (119.6 - 1255.6) | 0.04  (0.03 - 0.05) |  |
|  | **Other neurological disorders** |  |  |  |  |  |  |
|  | Deaths | 34103.3  (31527.8 - 36597.2) | 0.8  (0.7 - 0.8) | 59609.5  (55082.5 - 64550.6) | 0.8  (0.7 - 0.8) | 0.05  (-0.04 - 0.14) |  |
|  | DALYs | 2724964.5  (2242498.5 - 3282796.9) | 50.5  (42.5 - 60.3) | 4263389.5  (3458864.1 - 5174136.1) | 55.9  (45.2 - 68.3) | 0.41  (0.31 - 0.51) |  |
|  | **Supplemental table 2: Incidence, deaths, and DALYs of neurological disorders from 1990 to 2019, and EAPC of ASR by diseases.**  Other neurological disorders include muscular dystrophy, Huntington’s disease, and other less common neurological disorders. DALYs=disability-adjusted life years. ASR= age-standardized rates. EAPC= estimated annual percentage change. | | | | | |  |
